# Effect of Polishing on Electrochemical Behavior and Passive Layer Composition of Different Stainless Steels

**DOI:** 10.3390/ma13153402

**Published:** 2020-08-01

**Authors:** Krzysztof Rokosz, Grzegorz Solecki, Gregor Mori, Rainer Fluch, Marianne Kapp, Jouko Lahtinen

**Affiliations:** 1 Faculty of Mechanical Engineering, Koszalin University of Technology, Racławicka 15-17, PL 75-620 Koszalin, Poland; grzegorz.solecki.sg@gmail.com; 2 Department of General, Analytical and Physical Chemistry, Montanuniversitaet Leoben, A-8700 Leoben, Austria; 3 Voestalpine Böhler Edelstahl, 8605 Kapfenberg, Austria; rainer.fluch@voestalpine.com (R.F.); marianne.kapp@voestalpine.com (M.K.); 4 Department of Applied Physics, Aalto University, 02150 Espoo, Finland; jouko.lahtinen@aalto.fi

**Keywords:** stainless steels, passivity, electropolishing, mechanical polishing, electrochemical behavior, XPS

## Abstract

In the present paper, the effect of different polishing methods (mechanical and electrochemical) on passive layer chemistry and the corrosion behavior of stainless steels is investigated. It was found that CrNiMo austenites have a substantially better corrosion behavior than CrMnN ones. The nickel is enriched underneath the passive layer, while manganese tends to be enriched in the passive layer. It was also noted that immersion of manganese into an electrolyte preferentially causes its dissolution. It was found that high amounts of chromium (27.4%), molybdenum (3.3%), nickel (29.4%), with the addition of manganese (2.8%) after mechanical grinding, generates a better corrosion resistance than after electrochemical polishing. This is most likely because of the introduction of phosphates and sulfates into its structure, which is known for steels with a high amount of manganese. For highly alloyed CrNiMo steels, which do not contain a high amount of manganese, the addition of phosphates and/or sulphates via the electropolishing process results in a decrease in pitting corrosion resistance, which is also observed for high manganese steels. Electropolished samples show detrimental corrosion properties when compared to mechanically polished samples. This is attributed to substantial amounts of sulfate and phosphate from the electropolishing electrolyte present in the surface of the passive layer.

## 1. Introduction

To smoothen steel surfaces, mechanical polishing and electropolishing can be performed. Mechanical polishing can introduce cold deformation, residual stresses, and debris into the steel surface. On the other hand, electropolishing will dissolve more active sites, such as chromium poor segregations and nonmetallic inclusions, especially manganese sulfides. Therefore, it has often been found that electropolished steels have better corrosion properties than mechanically polished ones. Examples are given by Lee and Lai [1] in their investigation of the surface condition of stainless steel 316L by electropolishing in a sulfuric acid-phosphoric acid mixed electrolyte. Through electrochemical tests (polarization scans and electrochemical potentiokinetic reactivation (EPR) tests), they found a substantial increase in corrosion properties in sulfuric acid after electropolishing. Furthermore, Hryniewicz, Rokosz et al. [2,3,4,5] found a substantial improvement in the corrosion properties of AISI 304L and AISI 316L after electropolishing when tested in Ringer solution, 3% NaCl, and distilled water at 25 °C (aerated conditions). Most authors [6,7,8,9,10] investigated the effect of electropolishing on stainless steel 316L and characterized corrosion properties in various media. They conclude that electropolishing improves corrosion properties.

Ziemniak [11] comes to more diverse conclusions by considering papers of Perge, Robertson, Maekawa et al., Warzee et al., and Guinard et al. [12,13,14,15,16]. Electropolished stainless steel in water at temperatures below 350 °C shows better corrosion properties due to simultaneous oxide layer formation (outer layer ferrite or chromite rich and inner layer (chromite rich)), with the latter growing faster at cold worked, energy rich sites. At higher temperatures (>450 °C), when steam is present, short-circuiting at cold worked sites and enhanced chromium diffusion along grain boundaries favors corrosion resistance of mechanically polished surfaces over electropolished ones.

There are different alloying concepts of austenitic stainless steels. The CrNiMo austenitic stainless steels get their fcc microstructure mainly by alloying with nickel. Nickel in this case is the most noble alloying element in these steels. Therefore, it is depleted in the passive layer and enriched underneath the passive layer in the so-called interface between the passive layer and bulk alloy [17,18,19].

The second, less widely spread group of austenitic stainless steels are the CrMnN austenites, where manganese is the alloying element that stabilizes the fcc lattice. CrMnN has been widely investigated by Speidel and Uggowitzer [20,21] and later by Mori and his group [22,23]. Manganese in contradiction to nickel is less noble than iron and behaves similarly to chromium with its electrochemical potential. However, Manganese is not a passivating metal, consequently it is enriched in the passive layer, though does not contribute to an increased passivity like molybdenum.

The goal of the present paper is to compare mechanical and electrochemical polishing and their effect on passivity and pitting corrosion resistance of austenitic stainless steels. Not only the most widely investigated stainless steel (316L), but also steels with different chemical compositions and alloying concepts (CrNiMo austenites vs. CrMnN austenites) are part of this study.

## 2. Materials and Methods

Four austenitic stainless steels were investigated, two CrNiMo austenites and two CrMnN austenites (Table 1). They have different alloying contents yielding to different pitting resistance equivalent numbers (PREN—PREN is indicated in the footnote of Table 1). Throughout this work the less widespread PREN_Mn_ is used. A negative factor of 1 for the alloying element manganese is included in the PREN formula. This is necessary when investigating high-manganese and low-manganese stainless steels together in one work. The mechanical properties of the investigated materials are shown in Table 2. All materials were solution annealed and had a purely austenitic grain, free from precipitates. Grain size was between 80 and 200 µm for all materials.

Mechanical polishing was done with SiC abrasive papers from #120 up to #1000. Then, polished specimens were stored for 24 h in a desiccator for passive film formation. Electropolishing was done in a commercial H_2_SO_4_-H_3_PO_4_ electrolyte E269 from Poligrat. Current density was 500 mA/cm^2^ at a temperature of 60 °C. Polishing time was 4 minutes.

Pitting corrosion properties were determined in a conventional three-electrode cell. As counter and reference electrodes, a platinum sheet and saturated calomel electrode (SCE) with a potential of 241 mV vs. H were used, respectively. As electrolyte, a high chloride containing NaCl solution with 80,000 ppm C_Cl_^−^ at starting pH = 7 was used at a temperature of 80 °C. The scan rate was 200 mV/h (forward and reverse scans), and prior to polarization scans, open circuit potential was measured for 1 h. The start of the scanning was done from E_OCP_ −100 mV_SCE_ to the limit of current density at 1 mA/cm^2^ or a potential of 2000 mV_SCE_. Each specimen was tested a minimum three times.

The X-ray photoelectron spectroscopy (XPS) measurements were performed using a Kratos Axis Ultra system (Kratos Analytical Ltd, Manchester, UK), equipped with a monochromatic AlKα X-ray source. All measurements were performed using the analysis area 0.3 mm × 0.7 mm. The measurements were performed with a 40 eV pass energy and 0.1 eV energy step. Individual regions of C 1s, O 1s, P 2p, S 2p, Cr 2p, Mn 2p, Fe 2p, Ni 2p, and Mo 3d were recorded.

The depth profiling was done using 5 keV Ar+ ions with 10 mA ion current and rastering an area of 4 mm × 4 mm. The sputter rate was calibrated with a NBS Cr/Ni Reference 2135l and was equaled 91.6 s/nm.

## 3. Results

Polarization scans are shown in Figure 1, Figure 2, Figure 3 and Figure 4 and in Supplementary Materials in Figures S1–S8. In all Figures, the left image refers to mechanically polished samples and the right one to electropolished ones. It has to be pointed out that repeatability was good in all cases. Every experiment was done three times to prove repeatability.

**Figure 1:**
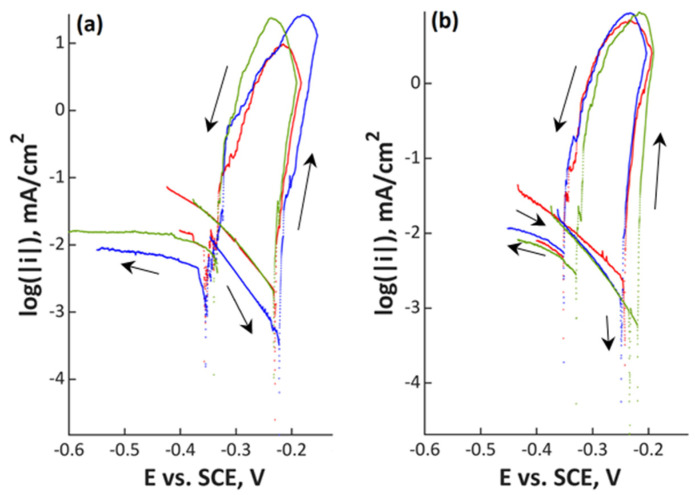
Polarization curves of (**a**) mechanically polished and (**b**) electropolished 18Cr21Mn2NiN stainless steel in aerated water solution with NaCl (80,000 ppm C_Cl–_, pH 7; 80 °C, 200 mV/h); three colors mean three measurements at the same point in the experiment plan.

The lowest alloyed material 18Cr21Mn2NiN (Figure 1) in both surface conditions demonstrated an open circuit potential of between –0.25 and –0.22 V_SCE_. There is no passive range for this steel in the investigated electrolyte and pitting starts immediately at the open circuit potential. The hysteresis in the curves shows that pitting occurred. Repassivation potential is in the range of –0.35 and –0.32 V_SCE_ independent of surface condition.

**Figure 2:**
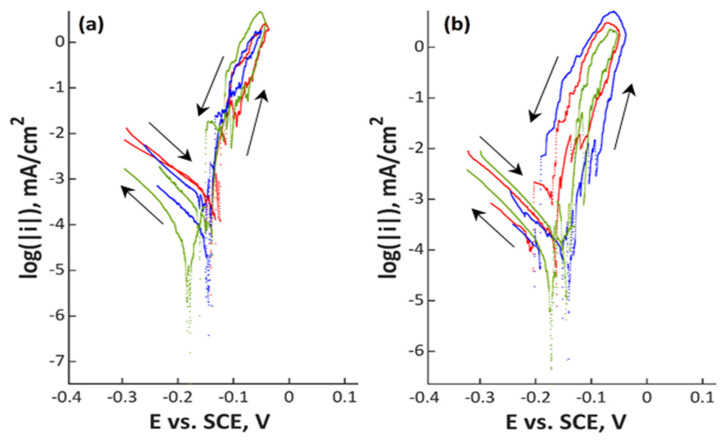
Polarization curves of (**a**) mechanically polished and (**b**) electropolished 20Cr20Mn7Ni2MoN stainless steel in aerated water solution with NaCl (80,000 ppm C_Cl–_, pH 7; 80 °C, 200 mV/h); three colors mean three measurements at the same point in the experiment plan.

Polarization curves of the higher alloyed manganese containing steel 20Cr20Mn7Ni2MoN are shown in Figure 2. The open circuit potential was between –0.16 and –0.14 V_SCE_, which is slightly more noble compared to the lower alloyed 18Cr21Mn2NiN. Again, there was no recorded passive region, though the repassivation potential shifted closer to the open circuit potential (from –0.20 up to –0.10 V_SCE_).

**Figure 3:**
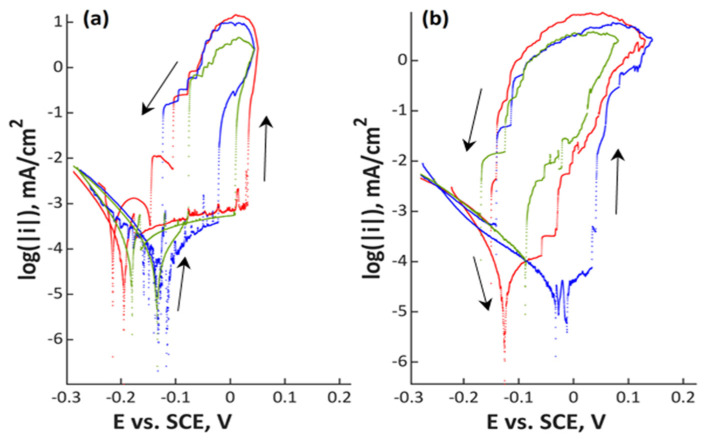
Polarization curves of (**a**) mechanically polished and (**b**) electropolished 18Cr15Ni3Mo stainless steel in aerated water solution with NaCl (80,000 ppm C_Cl–_, pH 7; 80 °C, 200 mV/h); three colors mean three measurements at the same point in the experiment plan.

**Figure 4:**
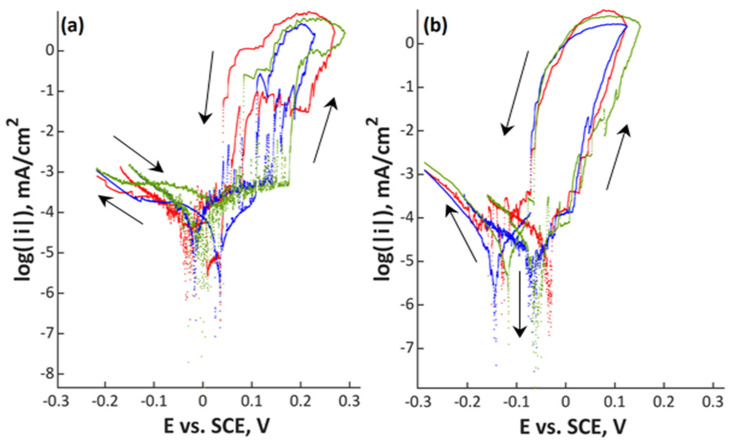
Polarization curves of (**a**) mechanically polished and (**b**) electropolished 27Cr29Ni3Mo stainless steel in aerated water solution with NaCl (80,000 ppm C_Cl–_, pH 7; 80 °C, 200 mV/h); three colors mean three measurements at the same point in the experiment plan.

In Figure 3 and Figure 4, the two stainless steels with low amounts of manganese are shown. Material 18Cr15Ni3Mo with an open circuit potential between −0.20 and −0.09 V_SCE_ showed a passive range with a width between 0.05 (electropolished) and 0.15 V (mechanically polished). This steel already showed a small difference between the mechanically and the electrochemically polished surface. The passive range was slightly wider for the mechanically polished condition. Repassivation potential was close to the open circuit potential for both specimens. The highest alloyed stainless steel 27Cr29Ni3Mo, a superaustenitic steel, showed the largest difference between the two investigated surface treatments. For the mechanically polished specimens, there was a clearly visible passive region, while it was not observed in the electrochemical-treated ones. The open circuit potential was close to 0 V_SCE_ for the mechanically polished samples, and it was slightly negative for the electropolished ones. A clear distinction was observed for the repassivation potentials: mechanically polished specimens repassivate at more noble potentials compared to the open circuit potential; electropolished ones repassivate close to the open circuit potential. Electrochemical parameters E_corr_, i_corr_, E_pit_, and E_rep_ are presented in Table 3 for a better overview of the data. 

**Figure 5:**
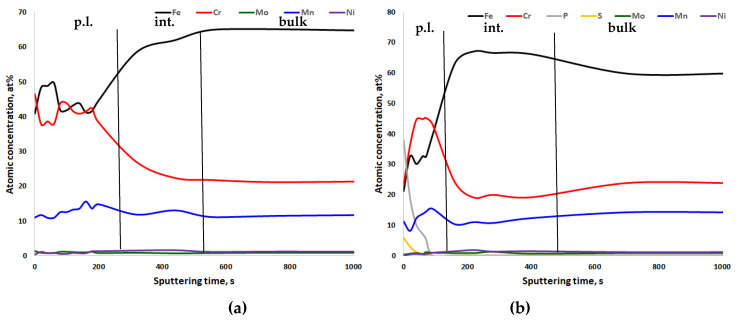
X-ray photoelectron spectroscopy (XPS) depth profiling results of the passive layer of (**a**) mechanically polished and (**b**) electropolished 18Cr21Mn2NiN stainless steel, 91.6 s ≈ 1 nm. p.l.—passive layer; int.—interface between passive layer and bulk metal; bulk—bulk metal.

**Figure 6:**
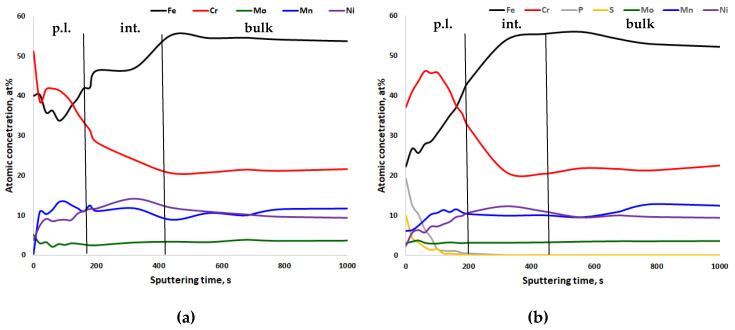
XPS depth profiling results of the passive layer of (**a**) mechanically polished and (**b**) electropolished 20Cr20Mn7Ni2MoN stainless steel, 91.6 s ≈ 1 nm. p.l.—passive layer; int.—nickel-enriched interface between passive layer and bulk metal; bulk—bulk metal.

**Figure 7:**
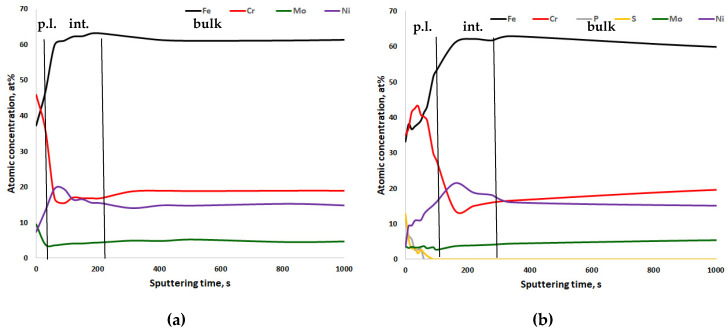
XPS depth profiling results of the passive layer of (**a**) mechanically polished and (**b**) electropolished 18Cr15Ni3Mo stainless steel, 91.6 s ≈ 1 nm. p.l.—passive layer; int.—nickel-enriched interface between passive layer and bulk metal; bulk—bulk metal.

**Figure 8:**
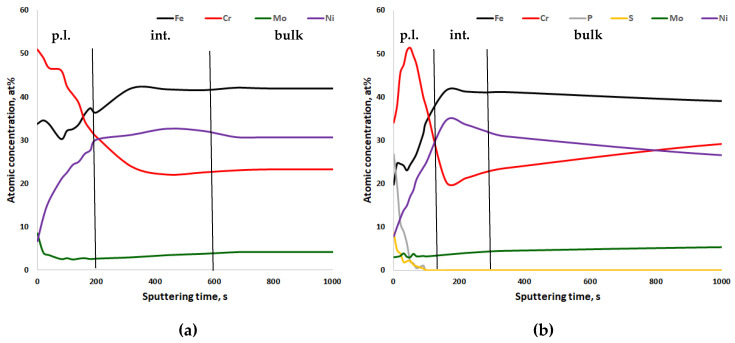
XPS depth profiling results of the passive layer of (**a**) mechanically polished and (**b**) electropolished 27Cr29Ni3Mo stainless steel, 91.6 s ≈ 1 nm. p.l.—passive layer; int.—nickel-enriched interface between passive layer and bulk metal; bulk—bulk metal.

**Figure 9:**
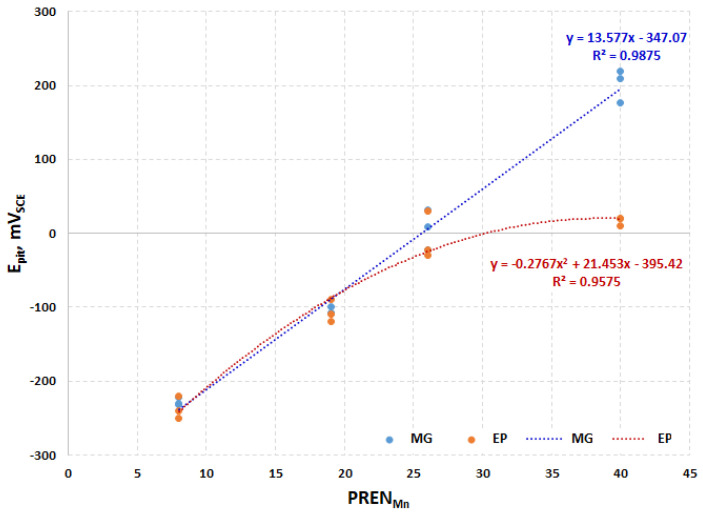
Comparison of pitting potential of studied stainless steels in function of modified pitting resistance equivalent numbers with Mn (PREN_Mn_), mechanically ground (MG), electropolished (EP).

In Figure 5, Figure 6, Figure 7 and Figure 8, the XPS analyses of the passive layers without oxygen are shown, and in Figures S9–S16 in the Supplementary Materials, the XPS analyses of the passive layers with oxygen of the four stainless steels after mechanical polishing and after electropolishing are shown. Additionally, in each figure, the width of the passive layer (p.l.) and the width of the interface (int.) between the passive layer and bulk metal is indicated by vertical lines. The passive layer thickness was determined via evaluation of the oxygen peak depth profile (not shown in the figures). The vertical line for the border between the passive layer and interface is placed where the oxygen content reaches the average between peak oxygen content at the surface and bulk oxygen content. XPS analysis always also gave some oxygen content in the bulk metal, which of course is not the case in reality. Therefore in Figure 6, Figure 7, Figure 8 and Figure 9, only metals and some electrolyte anions from electropolishing are presented. The elements included in these figures are normalized to 100%, excluding the oxygen content at any place. This was done to be able to better follow elemental enrichments in the passive layer and in the interface. The interface of CrNiMo austenites is known to be nickel enriched and the width of the nickel enrichment was determined as the “layer” between the passive layer surface and depth where the nickel content reaches the average between peak nickel content in the interface and bulk nickel content of the alloy. The interface was in contradiction to the passive layer no own phase, but only in terms of an enriched zone of nickel that was generated by selective oxidation of less noble elements.

Figure 5 shows the two XPS depth profiles of steel 18Cr21Mn2NiN. When considering the sputter rate of 91.6 s/nm, the passive layer of the mechanically polished specimen had a thickness of close to 6 nm (Figure 5a). The interface thickness was very close to that value. The electropolished specimen showed a slightly thinner passive layer and a thicker interface. No significant differences with respect to layer thicknesses were observed. In chemical composition, the electropolished specimen demonstrated a significant amount of P and S in the outer passive layer, up to a depth of close to 5.5 nm (Figure 5b). The passive layer was divided into an outer and an inner passive layer. The outer had a higher Fe/Cr ratio than the inner one. This was independent of the polishing process. Mn was slightly enriched in the passive layer.

The binding energy scale was calibrated to have the C1s peak at 284.8 eV, and all presented spectra were measured before the first sputtering. The reference binding energies related to Fe2p_3/2_, Cr2p_3/2_, P2p, S2p, and O1s are shown in Table S1 in Supplementary Materials. The peaks of oxygen (O1s), iron (Fe2p_3/2_), and chromium (Cr2p_3/2_) for mechanically treated samples are presented in Supplementary Materials in Figures S17–S19, respectively, while the oxygen (O1s), phosphorus (P2p), sulfur (S2p), iron (Fe2p_3/2_), and chromium (Cr2p_3/2_) peaks for electropolished ones are shown in Figures S20–S24 in Supplementary Materials, respectively. The analysis of XPS data indicates that the tops of the passive layers of all mechanically grinding samples consist mainly of oxides (Fe_2_O_3_, Cr_2_O_3_,) and hydroxides (FeOOH, CrOOH), which can be determined on the basis of the maxima of Fe2p_3/2_, Cr2p_3/2_, and O1s peaks at 710.8–711.4 eV, 576.7–576.9 eV, 530 eV (O^2−^), and 531.2–531.4 (OH^−^), respectively. The passive layers of electropolished steels consist of oxides (FeO, Fe_2_O_3_, Cr_2_O_3_) and hydroxides (FeOOH, CrOOH) with sulfates (FeSO_4_), which is indicated by the binding energies of oxygen (O1s: 531.4–531.5 eV), sulfur (S2p: 168.4–168.7 eV), iron (Fe2p_3/2_: 709.8–711.0 eV), and chromium (Cr2p_3/2_: 576.7–576.8 eV). The binding energies of phosphorus P2p equal to 133.1–133.2 eV clearly suggest that in the passive layer, there are phosphates of chromium and/or iron; however, this is not possible to conclude based on peak maximum analyses. In addition, it should be added that the C–O and O–C=O as well as water molecules (H_2_O) bondings could have an influence on the shape of O1s peaks.

Figure 6 shows the chemical composition of the surface profiles of steel 20Cr20Mn7Ni2MoN. The thicknesses of the passive layer and interface were similar to the ones for the steel 18Cr21Mn2NiN. This is easy to understand when considering that both alloys have very similar Cr and Mn contents. Only Ni is highly different between these two steels. Two clearly separated sublayers in the passive layer were not observed; however, the Cr content of the passive layer decreased towards the surface for the electropolished condition. Again the most distinct difference between the mechanically and electropolished specimens is the P and the S content of the electropolishing electrolyte (compare Figure 6b with Figure 6a). The passive layer demonstrated a certain amount of Mn enrichment close to the interface.

The passive layer and interface chemistry of conventional nickel austenite 18Cr15Ni3Mo is shown in Figure 7. The passive layer, independent of the polishing process, was thinner when compared to the above-discussed CrMnN steels. There was no significant change in the chemical composition of metal ions in the passive layer between differently polished specimens. Again there was a pronounced P and S peak close to the surface of the electropolished specimen. The nickel enrichment can be seen much more clearly due to the higher amount of nickel the steel contains.

The passive layer and interface chemistry of the highest alloyed stainless steel 27Cr29Ni3Mo is shown in Figure 8. Independent of the applied polishing process, the Cr content of the passive layer was highest, as compared to the other steels, due to the higher Cr content in the alloy. Again, the electropolished specimen had a pronounced P and S content in the outer passive layer (Figure 8b). The thickness of the interface of the mechanically polished specimen could not be determined exactly since there was no pronounced nickel enrichment (Figure 8a).

Interestingly, for the two mechanically polished CrNiMo austenites, there was an enrichment of Mo in the outer passive layer, and also Cr was enriched until the surface (Figure 8a and Figure 9a). This was not found in any other specimen.

## 4. Discussion

In the present paper, the effect of different polishing procedures on the passive layer chemistry and corrosion behavior of stainless steels, based on potentiodynamic corrosion and XPS measurements, is presented. It was found that CrNiMo austenitic stainless steels show a better corrosion than CrMnN ones. It was also noted that the pitting potential is a function of PREN_MN_, which is shown in Figure 9. For the three lower alloyed steels, surface treatment has no effect on pitting potential, while for the highest alloyed 27Cr29Ni3Mo, there is a substantial difference of almost 0.2 V (with the mechanically polished specimens being superior over electropolished ones). After the electropolishing process, the passive layers were less noble than the ones after mechanical treatments. This can be explained by the sulfates and phosphates from the electropolishing electrolyte, which are always present after this treatment in passive layers and were confirmed by XPS measurements. 

It was shown that the addition of molybdenum causes the formation of a compact layer, while manganese adds-on, negatively affecting its corrosion resistance. It was demonstrated that steels containing a lot of manganese (20–21%), low amounts of nickel (1.7–7%), and molybdenum (0.5–2.3%) with a PREN_Mn_ of less than 19 do not show differences in pitting corrosion resistance after mechanical and electrochemical treatments. In the case when the amount of manganese decreased (1.8–2.8%), with a simultaneous increase in nickel (14.7–29.4%) and molybdenum (2.8–3.3%), the pitting corrosion resistance of mechanically polished samples was significantly higher than that of the electrochemical ones. This phenomenon can be explained by the fact that a large addition of manganese in the steel structure probably has a negative effect on the pitting corrosion resistance of the passive layer, and any additional incorporation of phosphates and/or sulphates into the chromium–molybdenum structure has no significant influence on its corrosion resistance. In the case of steel with a high amount of chromium (27.4%), molybdenum (3.3%), nickel (29.4%), and a small amount of manganese (2.8%), after mechanical polishing, a stable and tight passive layer is formed, which is weakened by the introduction of phosphates and sulfates to its structures. From this, we can draw the conclusion that for high-alloyed steels that do not contain large amounts of manganese, the addition of phosphates and/or sulphates by electropolishing results in a decrease in the pitting corrosion resistance, similar to the one observed in the case of steels with high amounts of manganese.

In this discussion, the possible effect of nickel on pitting has thus far been neglected. Nickel usually is not included in PREN, so most authors believe that nickel has no effect. Speidel et al. [21,28,29,30,31] found nickel to have a small negative effect with respect to the pitting corrosion resistance, and they published this by proposing a measure of alloying as a resistance to corrosion (MARC) value. They also included the effects of carbon and manganese in MARC (Equation (1)).

MARC = %Cr + 3.3 × %Mo + 20 × %N + 20 × %C − 0.5 × %Mn− 0.25 × %Ni (1)

In contradiction to other authors, however, Speidel et al. attributed manganese with a rather small negative factor of -0.5. Some authors found -1 for manganese [26,27], and Jargelius-Pettersson found an even more negative factor for manganese (f_Mn_ = −1.6). Since all authors investigated different ranges of the chemical composition of stainless steels, all numbers have to be taken with care, especially when only one group of authors postulates a very small factor for a certain element, like Speidel does for nickel (f_Ni_ = −0.25).

It has to be considered that all PREN and MARC formulae are the result of regressions of experimental data heading for a (in most cases linear) relationship between chemical composition and critical pitting temperature. There is no physical explanation for PREN (or MARC), and a linear relation is unrealistic over a wide range of chemical compositions of steels. In addition, there is no obvious reason for nickel having a detrimental effect on pitting resistance, since nickel, as a noble alloying element, is enriched underneath the passive layer (also found in this work), and thus, nickel hinders the corrosive attack in an early pit that has just nucleated. Therefore, it is more likely that nickel has a positive effect by retarding acidification of the pit electrolyte. Nickel having a negative effect in PREN, therefore, is not realistic.

With respect to surface treatment, it can be concluded that electrochemical polishing causes the formation of phosphates and sulfates in the outer passive layer, which does not matter for the alloys 18Cr21Mn2NiN, 20Cr20Mn7Ni2MoN, 18Cr15Ni3Mo, but it matters for the highest alloyed steel 27Cr29Ni3Mo, in which, after mechanical treatment, the oxides/hydroxides of chromium and molybdenum in the passive layer are much more tight and compact. It was also found that the passive layers of CrNiMo austenites are thinner when compared to CrMnN austenites, which may be associated with a lower defect order in the passive layer (vacancies, lattice defects). In addition, it was found that nickel is enriched underneath the passive layer, while manganese tends to be enriched in the passive layer.

## 5. Conclusions

- CrNiMo austenites show a substantially better corrosion behavior than CrMnN austenites;
- For all steels, chromium enrichment was noted in the passive layers;
- For CrNiMo austenites, there is nickel enrichment underneath the passive layer, while for CrMnN austenites, manganese tends to be enriched in the passive layer;
- After electropolishing, sulfate and phosphate are present in the passive layers. The poor corrosion properties of electropolished specimens, as compared to mechanically ground ones, can be explained by this.

## Figures and Tables

**Table 1:** Chemical composition of investigated stainless steels, in wt.%.

Material	C	Si	Mn	Cr	Ni	Mo	N	PREN	PREN_Mn_
18Cr21Mn2NiN	<0.06	0.1	21.2	18.2	1.7	0.5	0.6	29	8
20Cr20Mn7Ni2MoN	<0.03		20.0	20.0	7.0	2.3	0.7	39	19
18Cr15Ni3Mo (S31603)	<0.03	0.3	1.8	17.5	14.7	2.8	0.1	28	26
27Cr29Ni3Mo (N08028)	<0.03	0.3	2.8	27.4	29.4	3.3	0.3	43	40

PREN = %Cr + 3.3 × %Mo + 16 × %N [24,25]. PREN_Mn_ = %Cr + 3.3 × %Mo + 16 × %N − 1 × %Mn [26,27].

**Table 2:** Mechanical properties of investigated stainless steels at room temperature.

Material	Yield Strength [MPa]	Tensile Strength [MPa]	Fracture Elongation [%]
18Cr21Mn2NiN	560	920	65
20Cr20Mn7Ni2MoN	510	900	54
18Cr15Ni3Mo (S31603)	290	560	65
27Cr29Ni3Mo (N08028)	380	790	64

**Table 3:** Summary of electrochemical parameters from polarization curves of investigated stainless steels in aerated water solution with NaCl (80,000 ppm C_Cl_^–^, pH 7; 80 °C, 200 mV/h).

Material	After Mechanical Grinding				After Electrochemical Polishing			
	E_corr_ mV_SCE_	i_corr_ mA/cm^2^	E_pit_ mV_SCE_	E_rep_ mV_SCE_	E_corr_ mV_SCE_	i_corr_ mA/cm^2^	E_pit_ mV_SCE_	E_rep_ mV_SCE_
18Cr21Mn2NiN	−230	8.21 × 10^−4^	−230	−331	−240	9.74 × 10^−4^	−240	−350
	−222	2.21 × 10^−4^	−222	−322	−250	5.13 × 10^−4^	−250	−350
	−232	8.21 × 10^−4^	−232	−329	−220	2.36 × 10^−4^	−220	−320
20Cr20Mn7Ni2MoN	−145	1.44 × 10^−4^	−100	−100	−160	1.06 × 10^−4^	−120	−200
	−138	6.82 × 10^−5^	−108	−108	−140	4.70 × 10^−5^	−90	−190
	−140	2.58 × 10^−4^	−108	−113	−140	7.58 × 10^−5^	−110	−130
18Cr15Ni3Mo (UNS S31603)	−195	5.13 × 10^−5^	32	−104	−130	2.26 × 10^−5^	−30	−150
	−111	1.79 × 10^−5^	−22	−124	−120	9.74 × 10^−6^	30	−140
	−179	5.13 × 10^−5^	9	−76	−90	4.72 × 10^−5^	−22	−130
27Cr29Ni3Mo (UNS N08028)	−32	1.23 × 10^−5^	219	41	−30	1.08 × 10^−5^	20	−70
	−97	1.90 × 10^−5^	209	−10	−60	5.13 × 10^−6^	20	−70
	−10	2.15 × 10^−5^	176	−82	−50	5.13 × 10^−6^	10	−60

## Supplementary Materials

The following are available online at https://www.mdpi.com/1996-1944/13/15/3402/s1; Figure S1. Polarization curves of passive layers of mechanically polished 18Cr21Mn2NiN stainless steel in aerated water solution with NaCl (80,000 ppm C_Cl–_, pH = 7; 80 °C, 200 mV/h); three colors mean three measurements at the same point in the experiment plan (a), one curve signifies the use of moving average (b); Figure S2. Polarization curves of passive layers of electropolished 18Cr21Mn2NiN stainless steel in aerated water solution with NaCl (80,000 ppm C_Cl–_, pH = 7; 80 °C, 200 mV/h); three colors mean three measurements at the same point in the experiment plan (a), one curve signifies the use of moving average (b); Figure S3. Polarization curves of passive layers of mechanically polished 20Cr20Mn7Ni2MoN stainless steel in aerated water solution with NaCl (80,000 ppm C_Cl–_, pH = 7; 80 °C, 200 mV/h); three colors mean three measurements at the same point in the experiment plan (a), one curve signifies the use of moving average (b); Figure S4. Polarization curves of passive layers of electropolished 20Cr20Mn7Ni2MoN stainless steel in aerated water solution with NaCl (80,000 ppm C_Cl–_, pH = 7; 80 °C, 200 mV/h); three colors mean three measurements at the same point in the experiment plan (a), one curve signifies the use of moving average (b); Figure S5. Polarization curves of passive layers of mechanically polished 18Cr15Ni3Mo stainless steel in aerated water solution with NaCl (80,000 ppm CCl–, pH = 7; 80 °C, 200 mV/h); three colors mean three measurements at the same point in the experiment plan (a), one curve signifies the use of moving average (b); Figure S6. Polarization curves of passive layers of electropolished 18Cr15Ni3Mo stainless steel in aerated water solution with NaCl (80,000 ppm CCl–, pH = 7; 80 °C, 200 mV/h); three colors mean three measurements at the same point in the experiment plan (a), one curve signifies the use of moving average (b); Figure S7. Polarization curves of passive layers of mechanically polished 27Cr29Ni3Mo stainless steel in aerated water solution with NaCl (80,000 ppm CCl–, pH = 7; 80 °C, 200 mV/h); three colors mean three measurements at the same point in the experiment plan (a), one curve signifies the use of moving average (b); Figure S8. Polarization curves of passive layers of electropolished 27Cr29Ni3Mo stainless steel in aerated water solution with NaCl (80,000 ppm CCl–, pH = 7; 80 °C, 200 mV/h); three colors mean three measurements at the same point in the experiment plan (a), one curve signifies the use of moving average (b); Figure S9. XPS depth profiling results of the passive layer of mechanically polished 18Cr21Mn2NiN stainless steel, 91.6 s 1 nm; Figure S10. XPS depth profiling results of the passive layer of electropolished 18Cr21Mn2NiN stainless steel, 91.6 s 1 nm; Figure S11. XPS depth profiling results of the passive layer of mechanically polished 20Cr20Mn7Ni2MoN stainless steel, 91.6 s 1 nm; Figure S12. XPS depth profiling results of the passive layer of electropolished 20Cr20Mn7Ni2MoN stainless steel, 91.6 s 1 nm; Figure S13. XPS depth profiling results of the passive layer of mechanically polished 18Cr15Ni3Mo stainless steel, 91.6 s 1 nm; Figure S14. XPS depth profiling results of the passive layer of electropolished 18Cr15Ni3Mo stainless steel, 91.6 s 1 nm; Figure S15. XPS depth profiling results of the passive layer of mechanically polished 27Cr29Ni3Mo stainless steel, 91.6 s 1 nm; Figure S16. XPS depth profiling results of the passive layer of electropolished 27Cr29Ni3Mo stainless steel, 91.6 s 1 nm; Figure S17. Oxygen O1s XPS spectra of the passive layer of mechanically ground stainless steels: (a) 18Cr21Mn2NiN, (b) 20Cr20Mn7Ni2MoN, (c) 18Cr15Ni3Mo, (d) 27Cr29Ni3Mo; Figure S18. Iron Fe2p3/2 XPS spectra of the passive layer of mechanically ground stainless steels: (a) 18Cr21Mn2NiN, (b) 20Cr20Mn7Ni2MoN, (c) 18Cr15Ni3Mo, (d) 27Cr29Ni3Mo; Figure S19. Chromium Cr2p3/2 XPS spectra of passive layer of mechanically ground stainless steels: (a) 18Cr21Mn2NiN, (b) 20Cr20Mn7Ni2MoN, (c) 18Cr15Ni3Mo, (d) 27Cr29Ni3Mo; Figure S20. Oxygen O1s XPS spectra of the passive layer of electropolished stainless steels: (a) 18Cr21Mn2NiN, (b) 20Cr20Mn7Ni2MoN, (c) 18Cr15Ni3Mo, (d) 27Cr29Ni3Mo; Figure S21. Phosphorus P2p XPS spectra of the passive layer of electropolished stainless steels: (a) 18Cr21Mn2NiN, (b) 20Cr20Mn7Ni2MoN, (c) 18Cr15Ni3Mo, (d) 27Cr29Ni3Mo; Figure S22. Sulphur S2p XPS spectra of the passive layer of electropolished stainless steels: (a) 18Cr21Mn2NiN, (b) 20Cr20Mn7Ni2MoN, (c) 18Cr15Ni3Mo, (d) 27Cr29Ni3Mo; Figure S23. Iron Fe2p_3/2_ XPS spectra of the passive layer of electropolished stainless steels: (a) 18Cr21Mn2NiN, (b) 20Cr20Mn7Ni2MoN, (c) 18Cr15Ni3Mo, (d) 27Cr29Ni3Mo; Figure S24. Chromium Cr2p_3/2_ XPS spectra of the passive layer of electropolished stainless steels: (a) 18Cr21Mn2NiN, (b) 20Cr20Mn7Ni2MoN, (c) 18Cr15Ni3Mo, (d) 27Cr29Ni3Mo; Table S1. Binding energies (BE, eV) of iron, chromium compounds
