# Supplementary material for: Effect of Polishing on Electrochemical Behavior and Passive Layer Composition of Different Stainless Steels
Source: Materials (Basel). 2020 Aug 1;13(15):3402. doi: 10.3390/ma13153402 (PMC7435799; doi:10.3390/ma13153402)
Supplement: Supplementary file 1 [file materials-13-03402-s001.pdf]

materials

Article

Effect of Polishing on Electrochemical Behavior and Passive Layer Composition of Different Stainless Steels

Supplementary files:

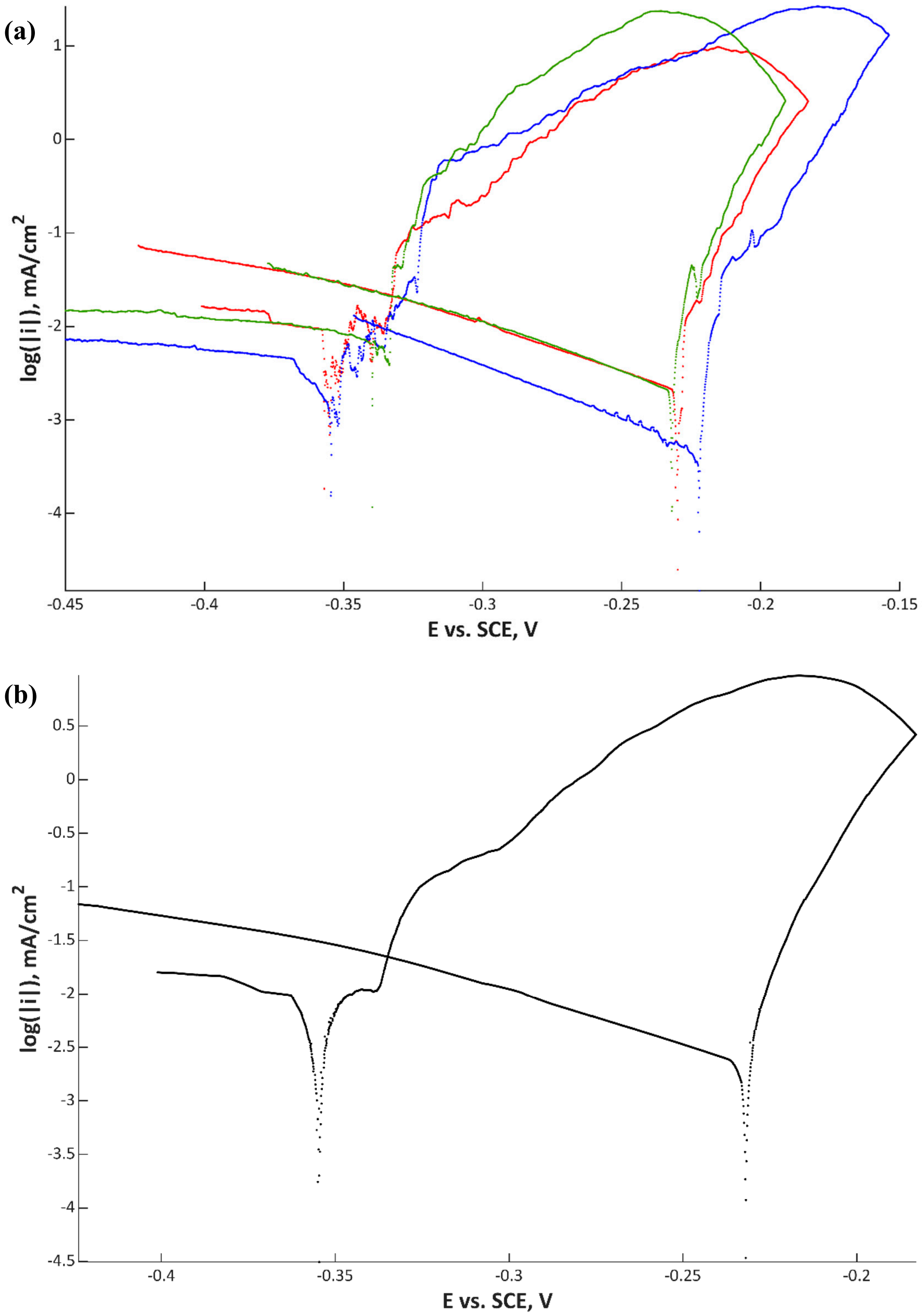

Figure S1. Polarization curves of passive layers of mechanically polished 18Cr21Mn2NiN stainless steel in aerated water solution with NaCl (80000 ppm C_{Cl^-}, pH = 7; 80 °C, 200 mV/h); three colors mean three measurements at the same point in the experiment plan (**a**), one curve signifies the use of moving average (**b**).

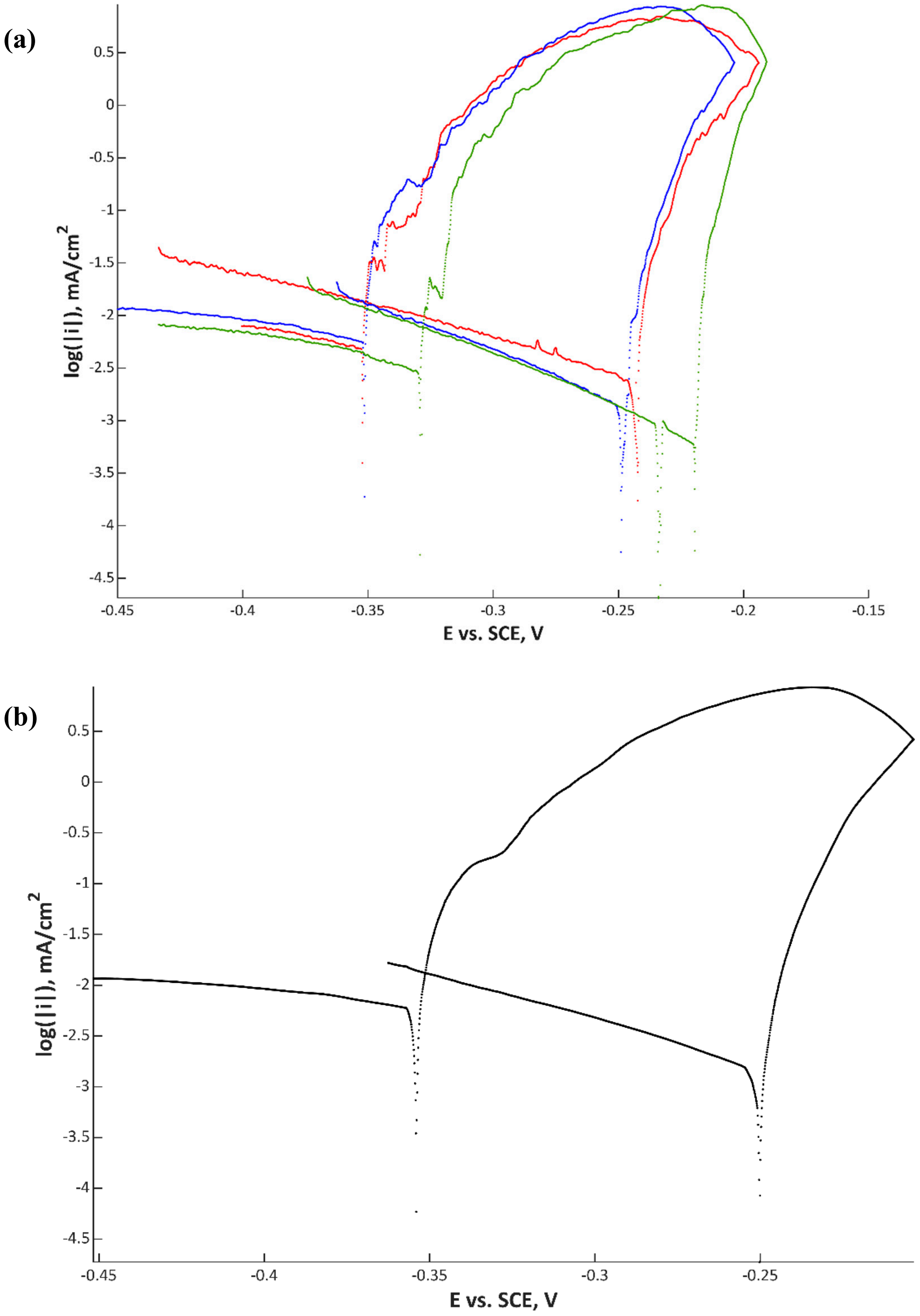

Figure S2. Polarization curves of passive layers of electropolished 18Cr21Mn2NiN stainless steel in aerated water solution with NaCl (80000 ppm C_{Cl^-}, pH = 7; 80 °C, 200 mV/h); three colors mean three measurements at the same point in the experiment plan (**a**), one curve signifies the use of moving average (**b**).

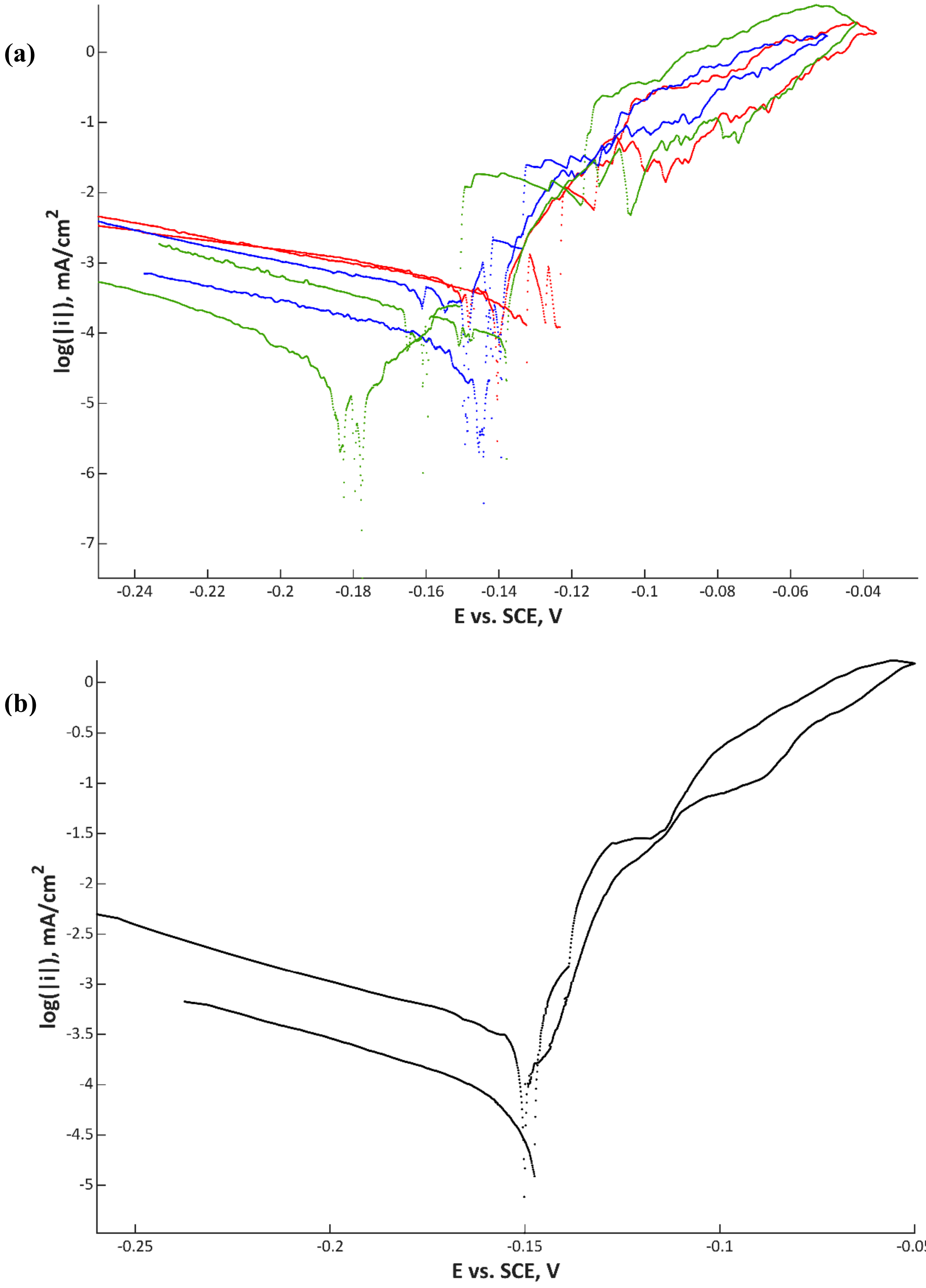

Figure S3. Polarization curves of passive layers of mechanically polished 20Cr20Mn7Ni2MoN stainless steel in aerated water solution with NaCl (80000 ppm C_{Cl^-}, pH = 7; 80 °C, 200 mV/h); three colors mean three measurements at the same point in the experiment plan (**a**), one curve signifies the use of moving average (**b**).

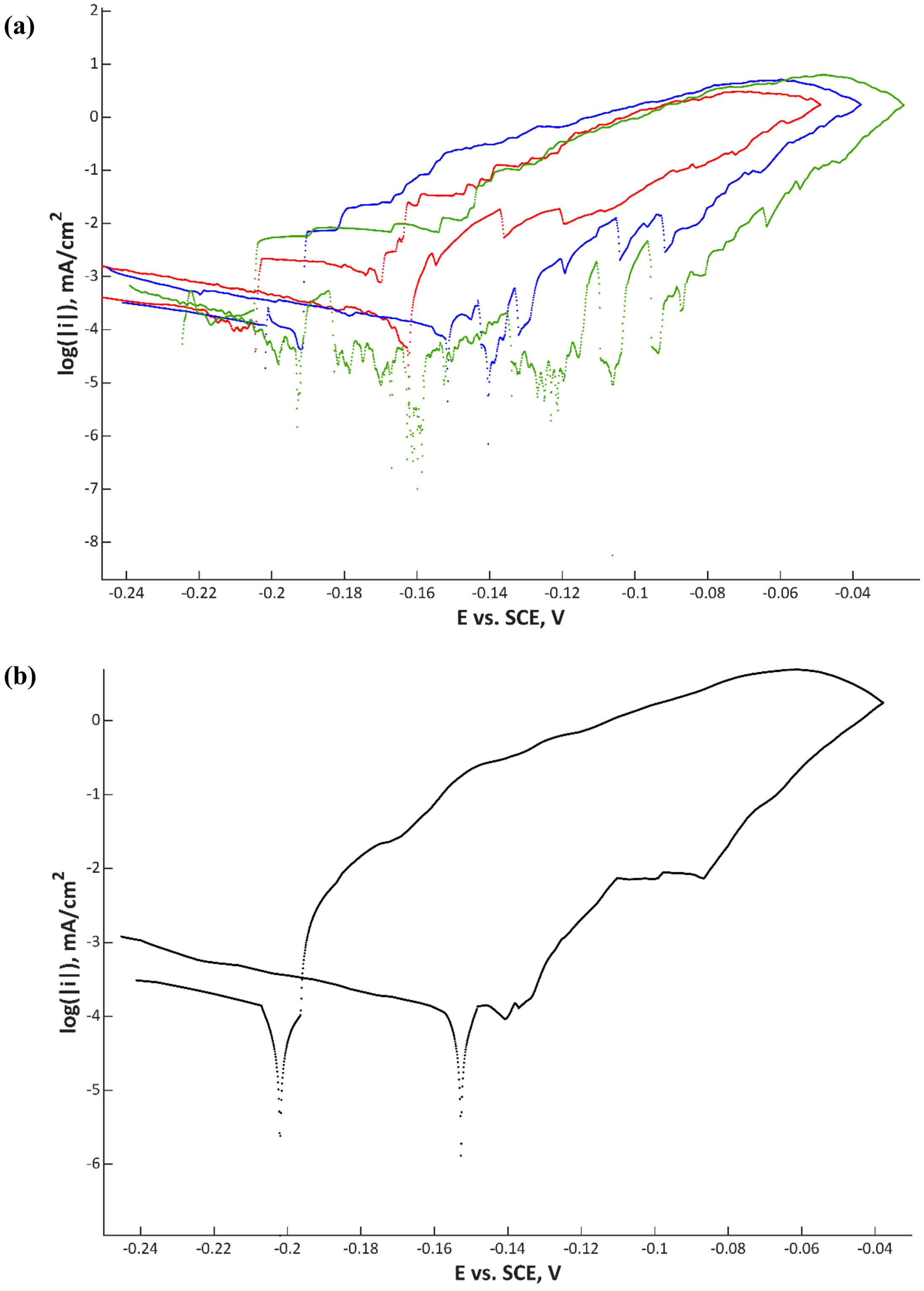

Figure S4. Polarization curves of passive layers of electropolished 20Cr20Mn7Ni2MoN stainless steel in aerated water solution with NaCl (80000 ppm C_{Cl^-}, pH = 7; 80 °C, 200 mV/h); three colors mean three measurements at the same point in the experiment plan (**a**), one curve signifies the use of moving average (**b**).

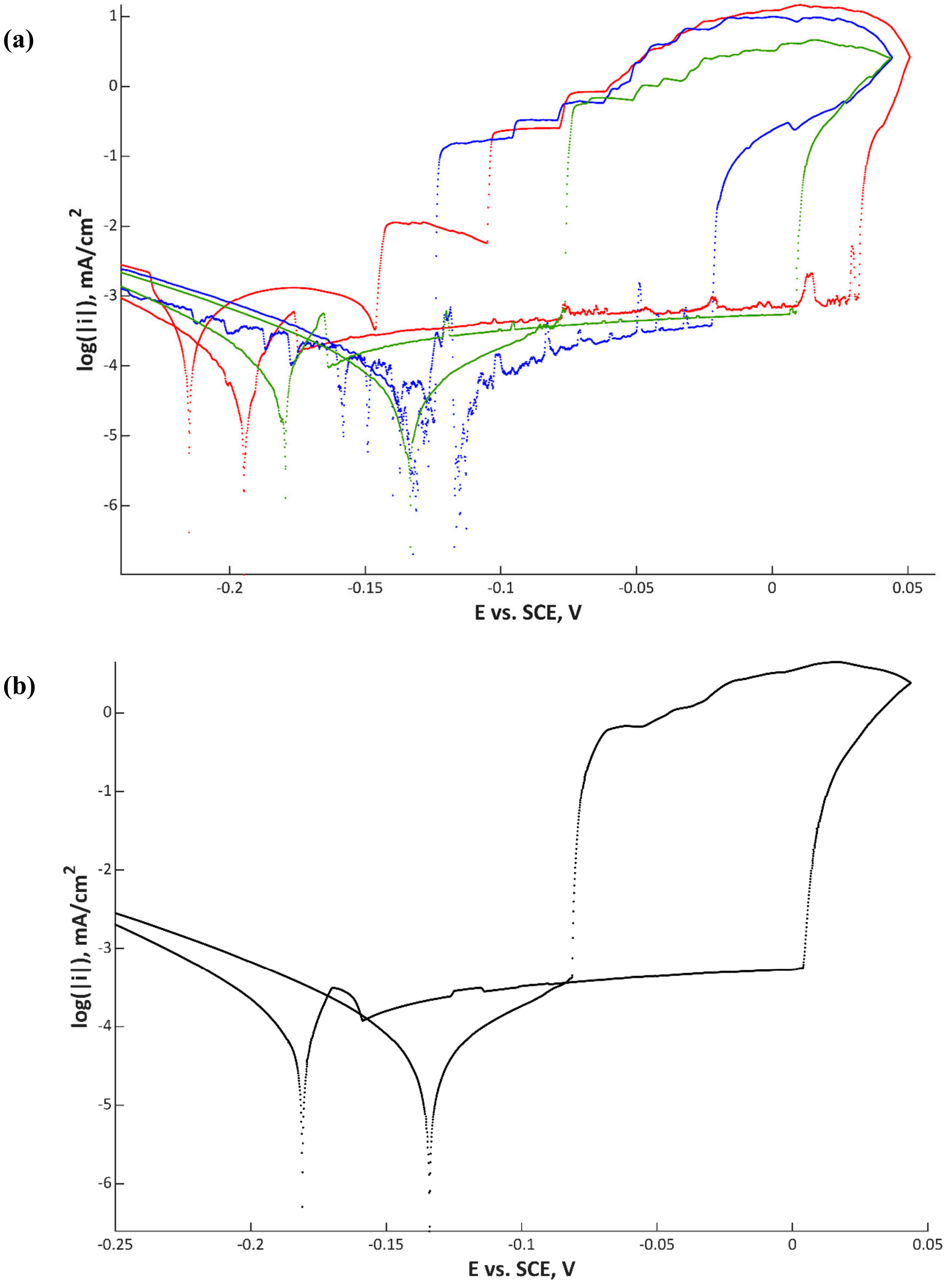

Figure S5. Polarization curves of passive layers of mechanically polished 18Cr15Ni3Mo stainless steel in aerated water solution with NaCl (80000 ppm C_{Cl^-}, pH = 7; 80 °C, 200 mV/h); three colors mean three measurements at the same point in the experiment plan (**a**), one curve signifies the use of moving average (**b**).

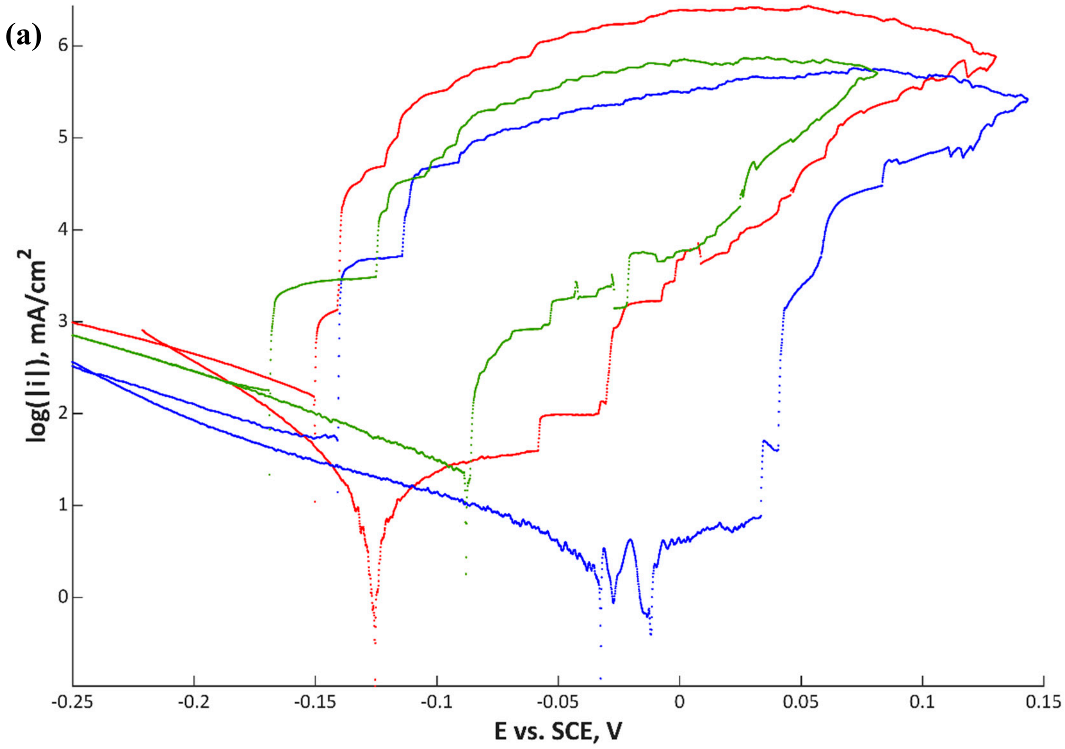

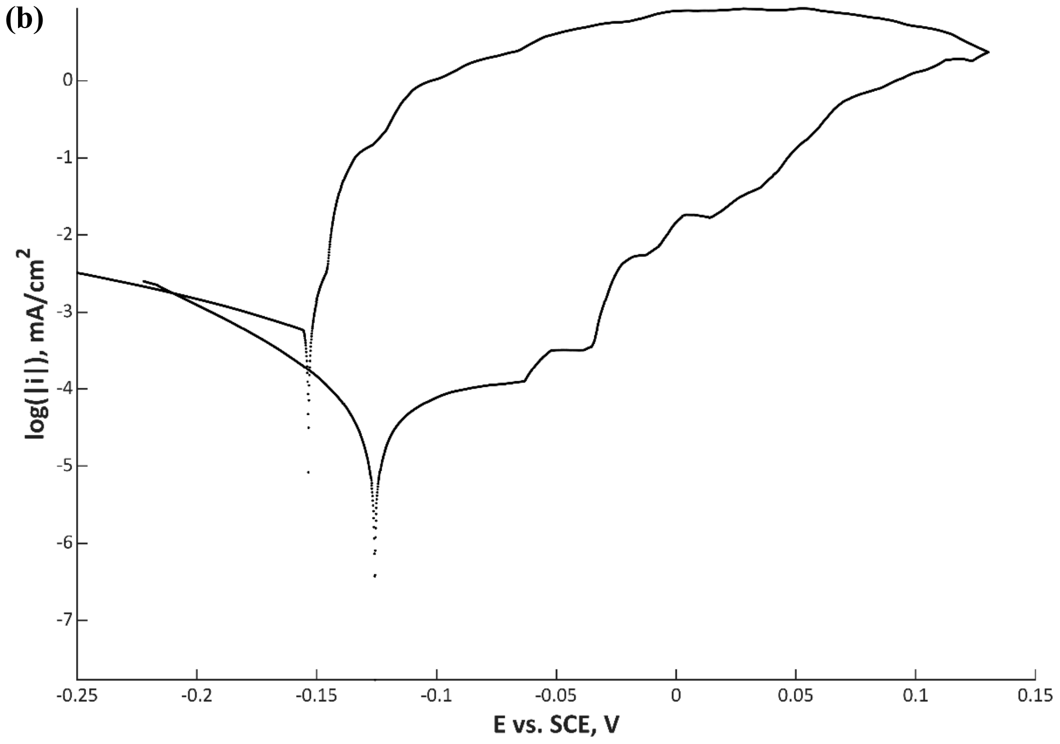

Figure S6. Polarization curves of passive layers of electropolished 18Cr15Ni3Mo stainless steel in aerated water solution with NaCl (80000 ppm C_{Cl^-}, pH = 7; 80 °C, 200 mV/h); three colors mean three measurements at the same point in the experiment plan (**a**), one curve signifies the use of moving average (**b**).

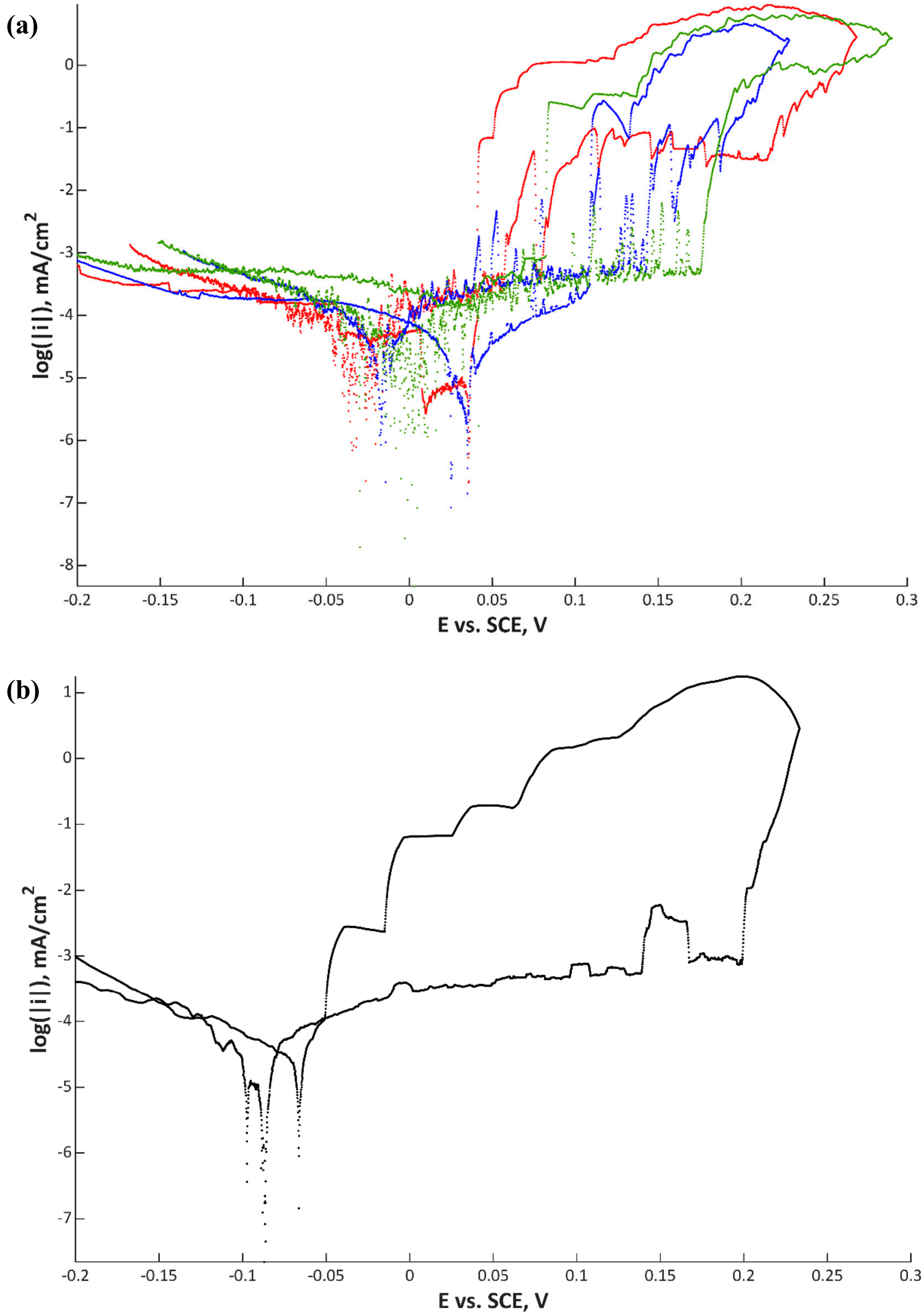

Figure S7. Polarization curves of passive layers of mechanically polished 27Cr29Ni3Mo stainless steel in aerated water solution with NaCl (80000 ppm C_{Cl^-}, pH = 7; 80 °C, 200 mV/h); three colors mean three measurements at the same point in the experiment plan (**a**), one curve signifies the use of moving average (**b**).

(a)

(b)

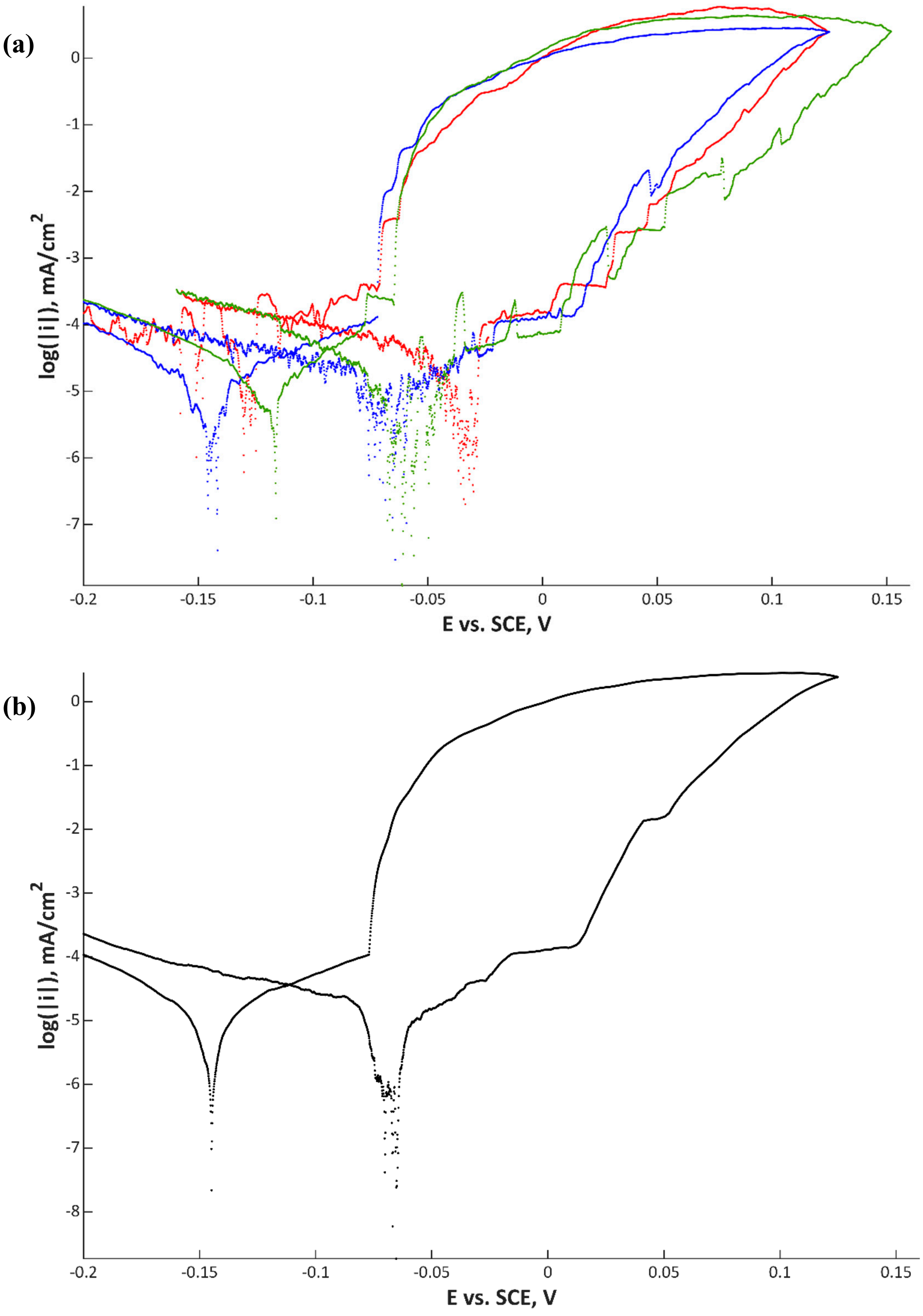

Figure S8. Polarization curves of passive layers of electropolished 27Cr29Ni3Mo stainless steel in aerated water solution with NaCl (80000 ppm C_{Cl^-}, pH = 7; 80 °C, 200 mV/h); three colors mean three measurements at the same point in the experiment plan (**a**), one curve signifies the use of moving average (**b**).

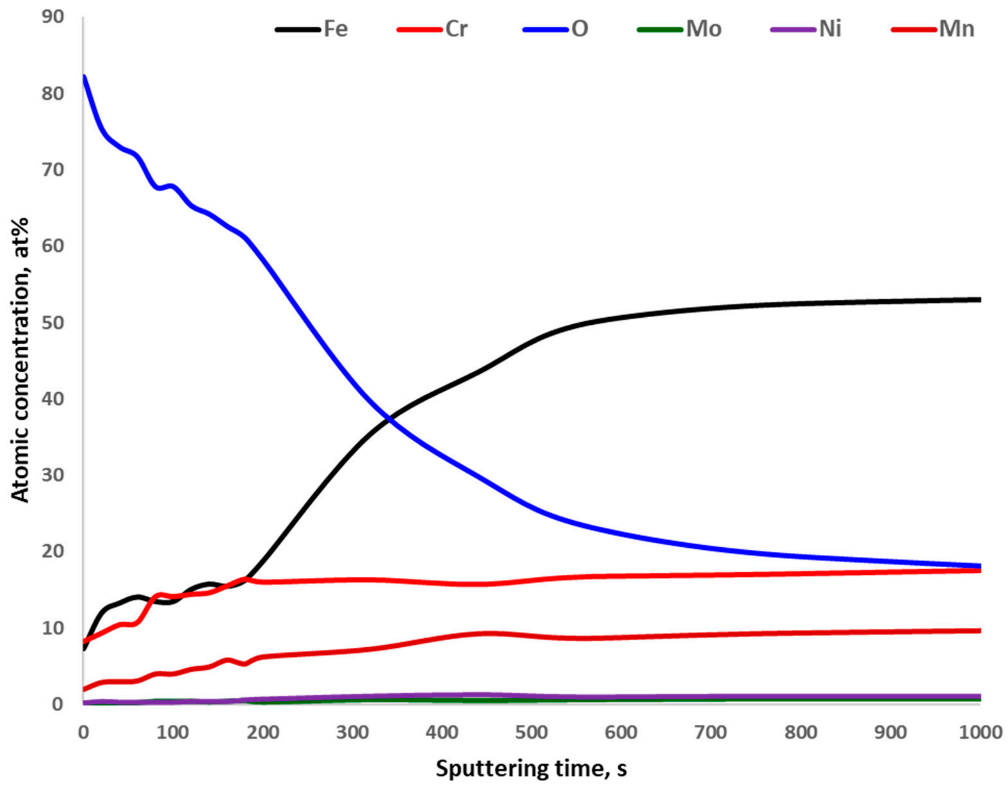

Figure S9. XPS depth profiling results of the passive layer of mechanically polished 18Cr21Mn2NiN stainless steel, 91.6 s ≈ 1 nm.

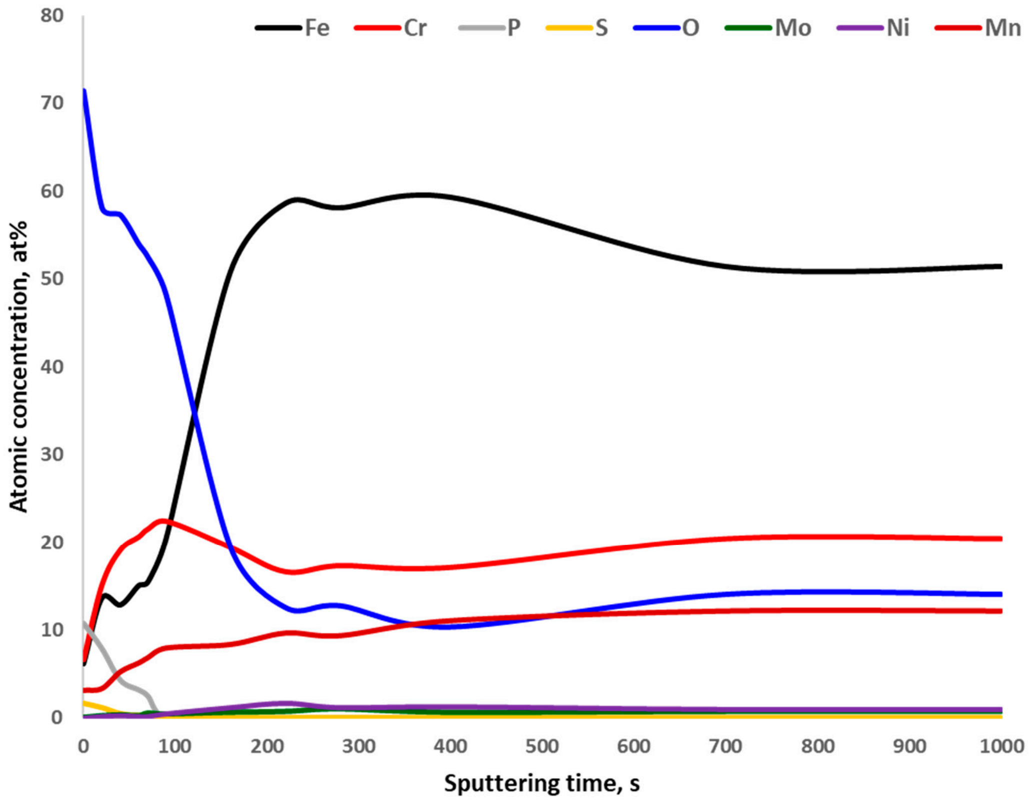

Figure S10. XPS depth profiling results of the passive layer of electropolished 18Cr21Mn2NiN stainless steel, 91.6 s ≈ 1 nm.

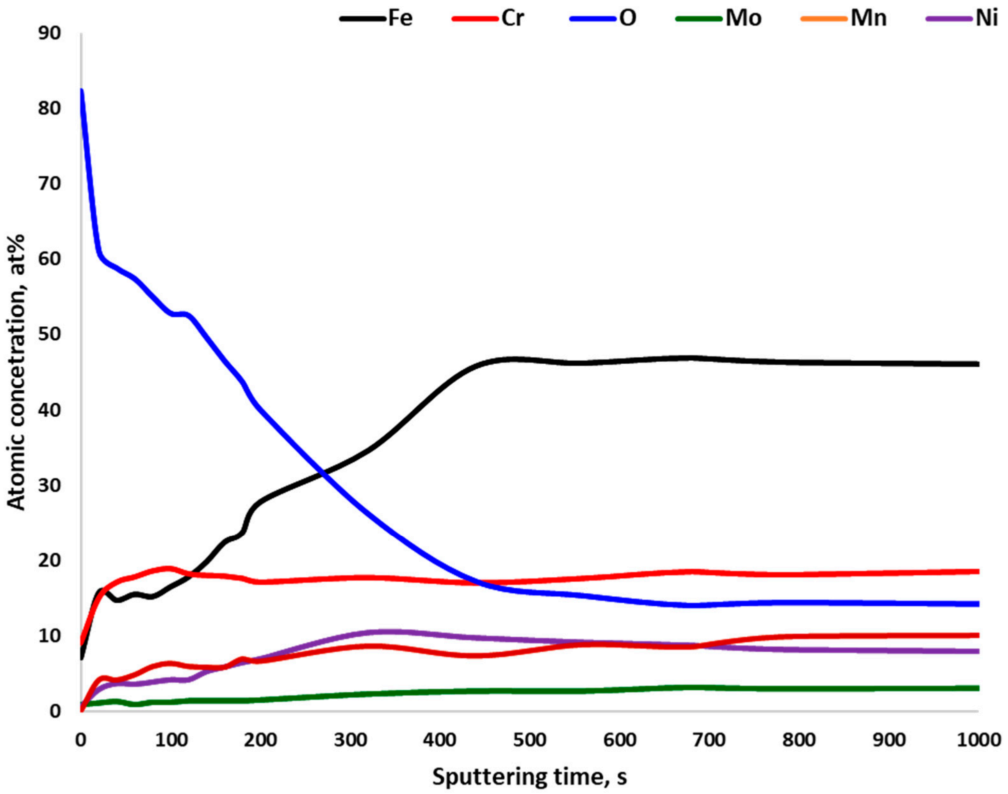

Figure S11. XPS depth profiling results of the passive layer of mechanically polished 20Cr20Mn7Ni2MoN stainless steel, 91.6 s ≈ 1 nm.

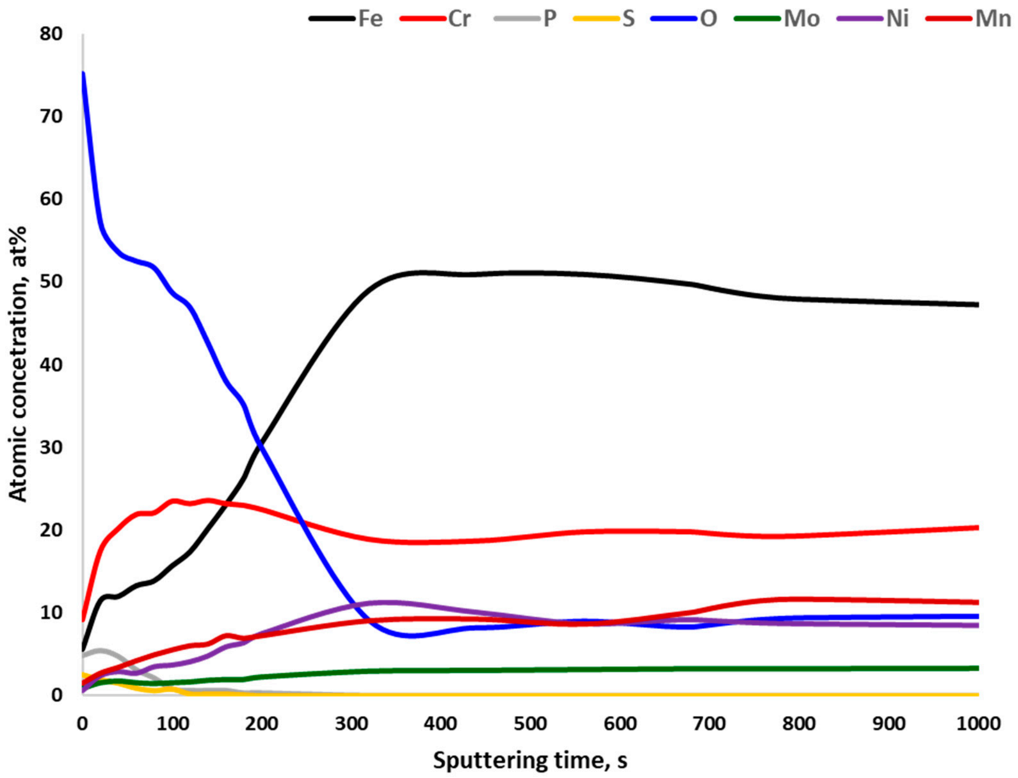

Figure S12. XPS depth profiling results of the passive layer of electropolished 20Cr20Mn7Ni2MoN stainless steel, 91.6 s ≈ 1 nm.

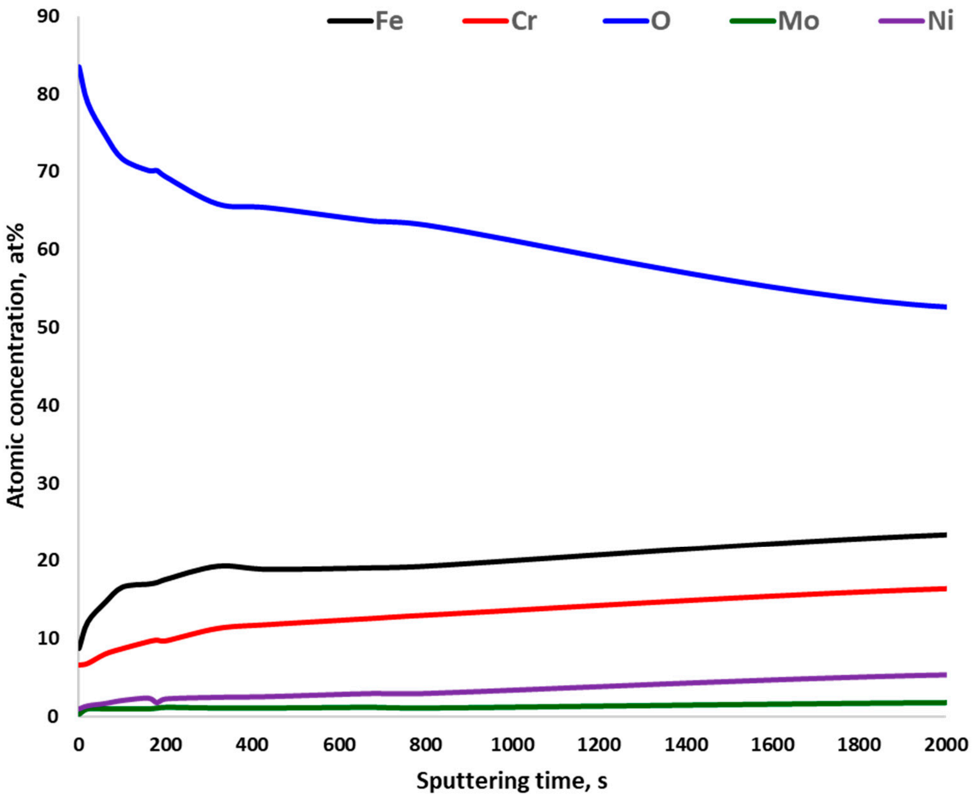

Figure S13. XPS depth profiling results of the passive layer of mechanically polished 18Cr15Ni3Mo stainless steel, 91.6 s ≈ 1 nm.

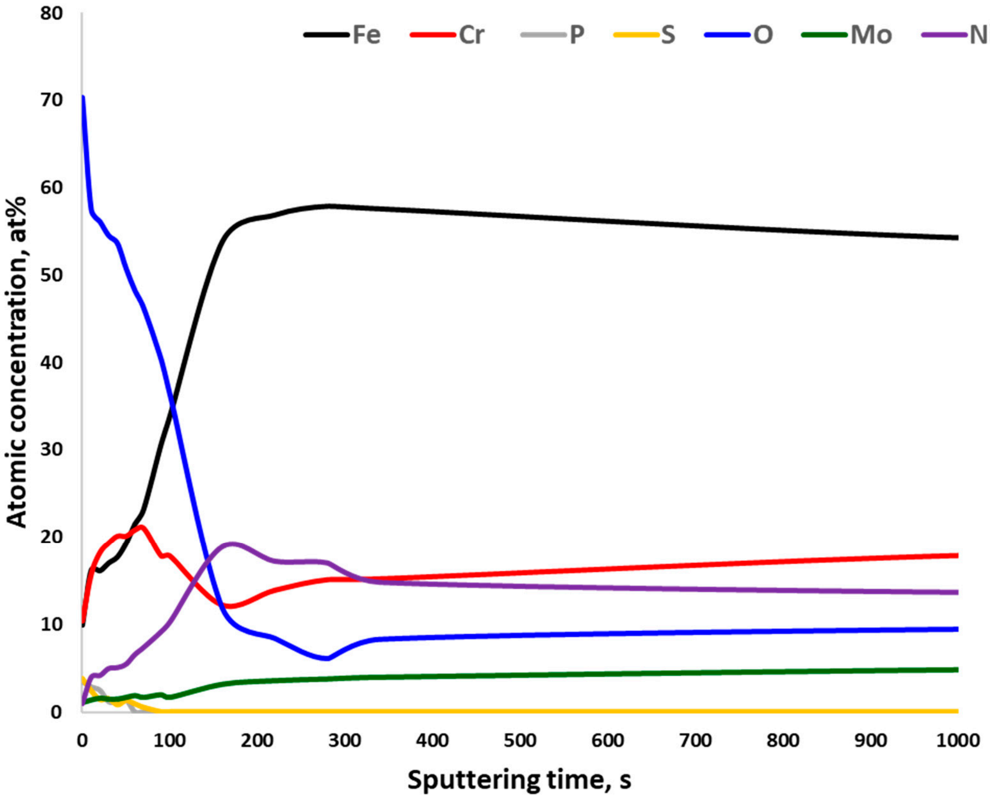

Figure S14. XPS depth profiling results of the passive layer of electropolished 18Cr15Ni3Mo stainless steel, 91.6 s ≈ 1 nm.

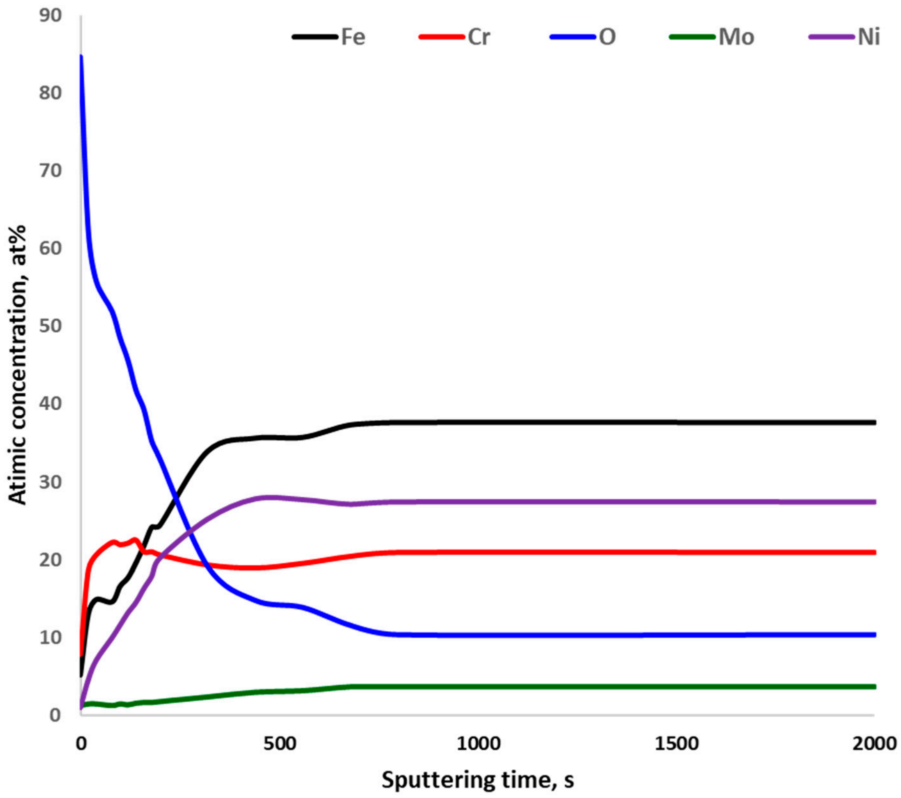

Figure S15. XPS depth profiling results of the passive layer of mechanically polished 27Cr29Ni3Mo stainless steel, 91.6 s ≈ 1 nm.

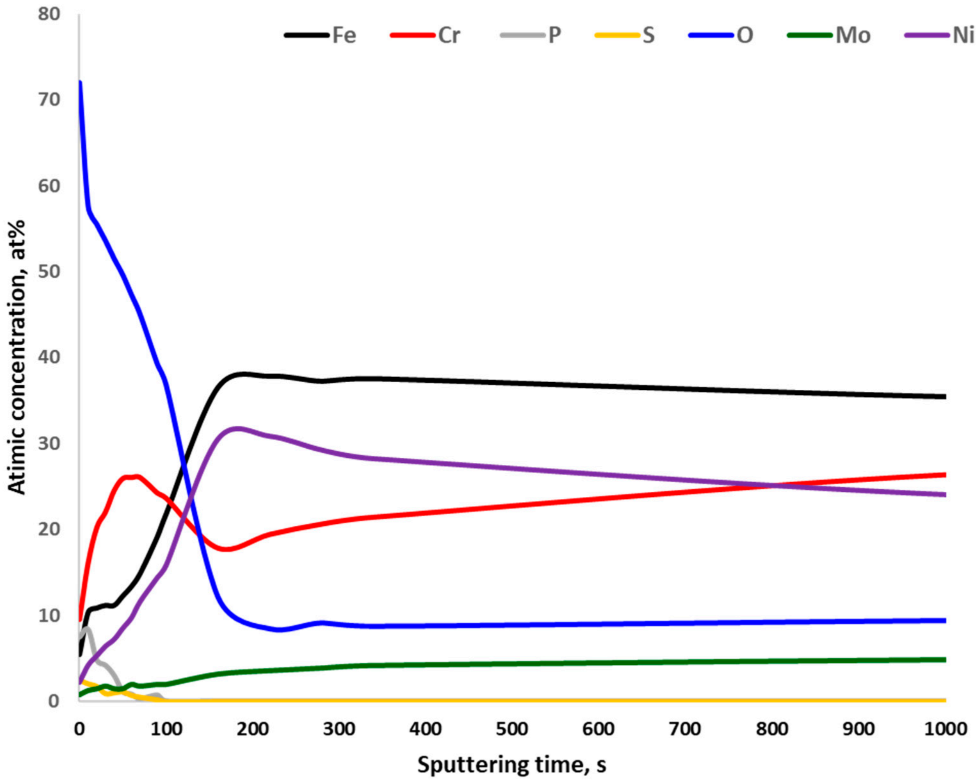

Figure S16. XPS depth profiling results of the passive layer of electropolished 27Cr29Ni3Mo stainless steel, 91.6 s ≈ 1 nm.

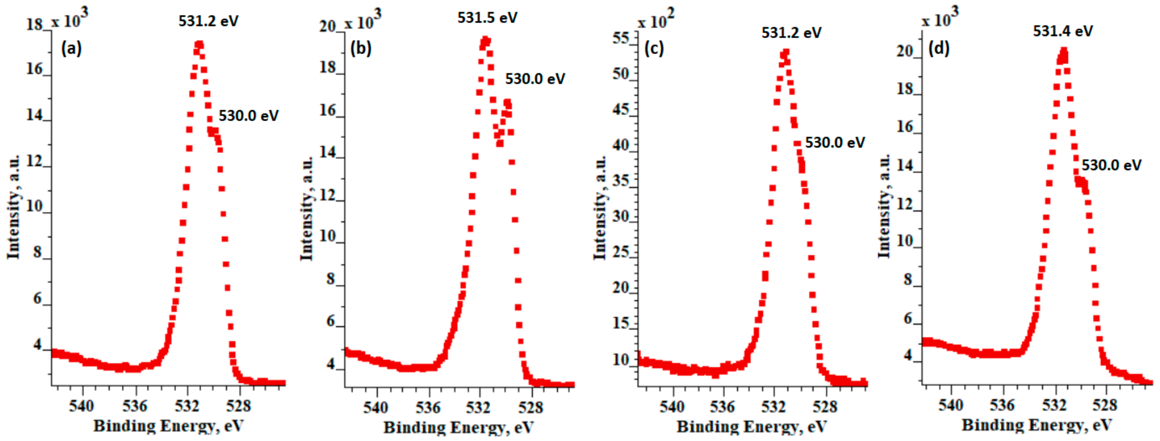

Figure S17. Oxygen O1s XPS spectra of the passive layer of mechanically ground stainless steels: (**a**) 18Cr21Mn2NiN, (**b**) 20Cr20Mn7Ni2MoN, (**c**) 18Cr15Ni3Mo, (**d**) 27Cr29Ni3Mo.

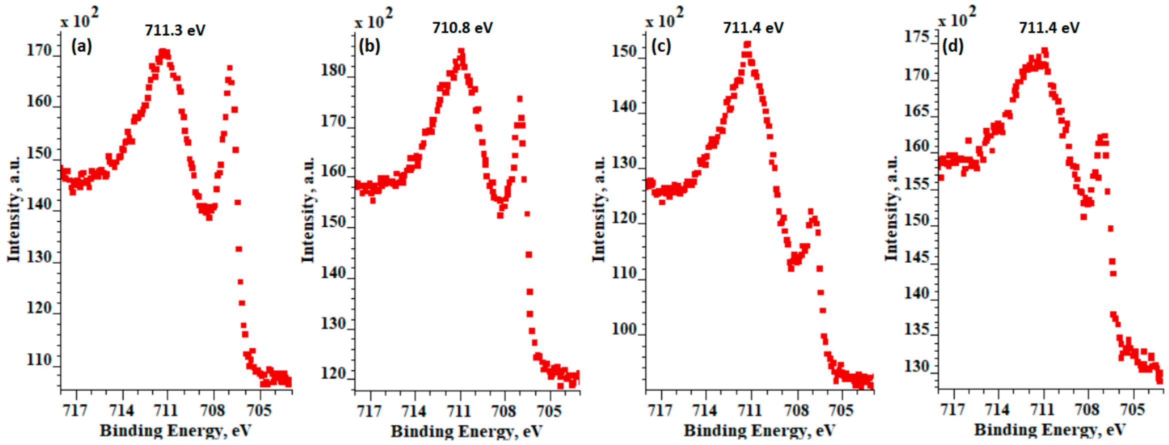

Figure S18. Iron Fe2p$_{3/2}$ XPS spectra of the passive layer of mechanically ground stainless steels: (**a**) 18Cr21Mn2NiN, (**b**) 20Cr20Mn7Ni2MoN, (**c**) 18Cr15Ni3Mo, (**d**) 27Cr29Ni3Mo.

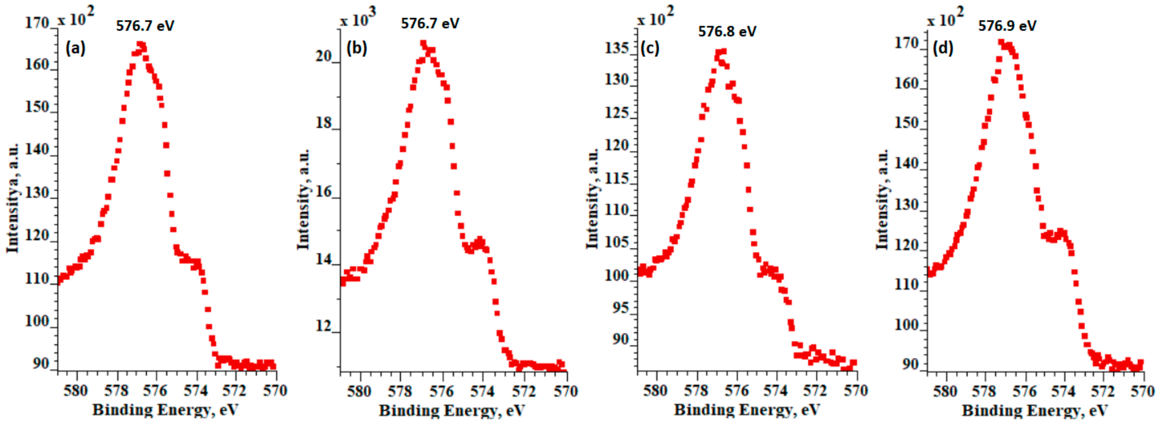

Figure S19. Chromium Cr2p$_{3/2}$ XPS spectra of passive layer of mechanically ground stainless steels: (**a**) 18Cr21Mn2NiN, (**b**) 20Cr20Mn7Ni2MoN, (**c**) 18Cr15Ni3Mo, (**d**) 27Cr29Ni3Mo.

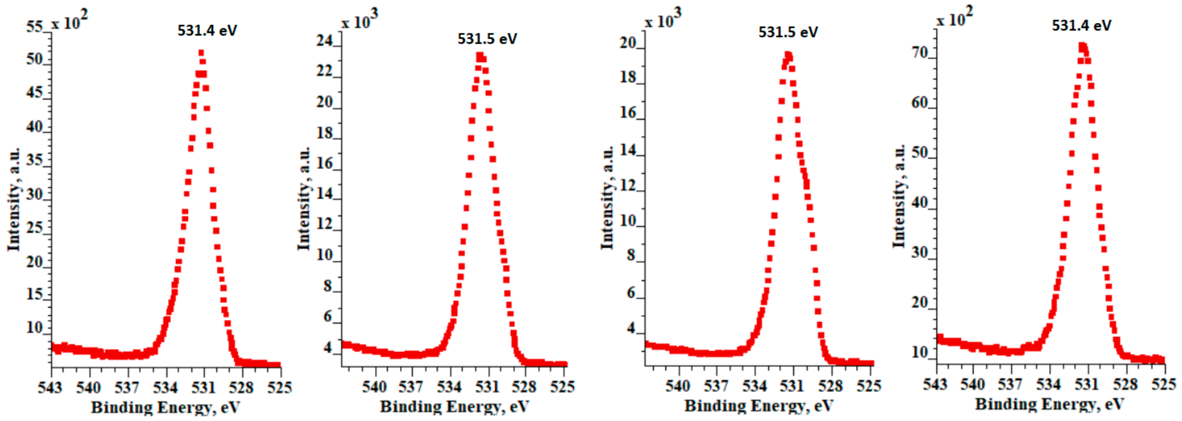

Figure S20. Oxygen O1s XPS spectra of the passive layer of electropolished stainless steels: (a) 18Cr21Mn2NiN, (**b**) 20Cr20Mn7Ni2MoN, (**c**) 18Cr15Ni3Mo, (**d**) 27Cr29Ni3Mo.

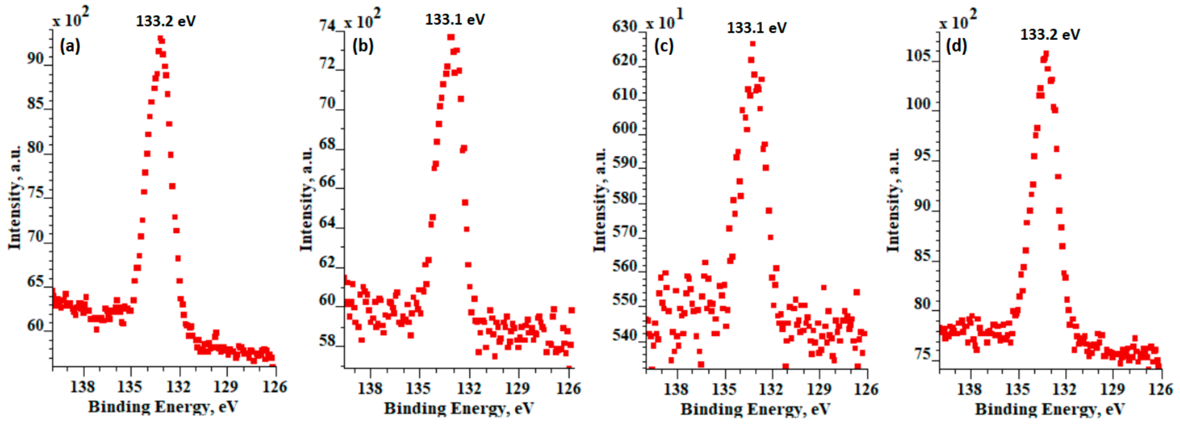

Figure S21. Phosphorus P2p XPS spectra of the passive layer of electropolished stainless steels: (**a**) 18Cr21Mn2NiN, (**b**) 20Cr20Mn7Ni2MoN, (**c**) 18Cr15Ni3Mo, (**d**) 27Cr29Ni3Mo.

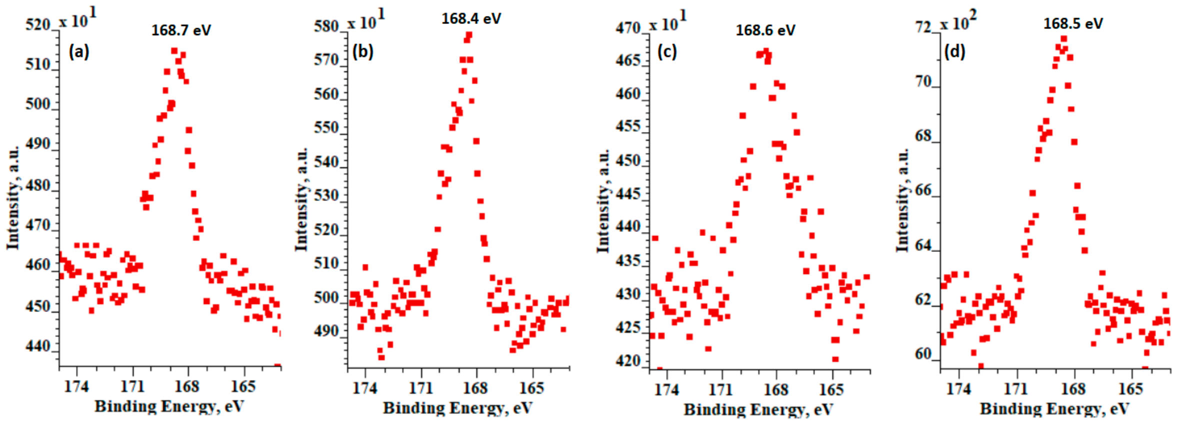

Figure S22. Sulphur S2p XPS spectra of the passive layer of electropolished stainless steels: (**a**) 18Cr21Mn2NiN, (**b**) 20Cr20Mn7Ni2MoN, (**c**) 18Cr15Ni3Mo, (**d**) 27Cr29Ni3Mo.

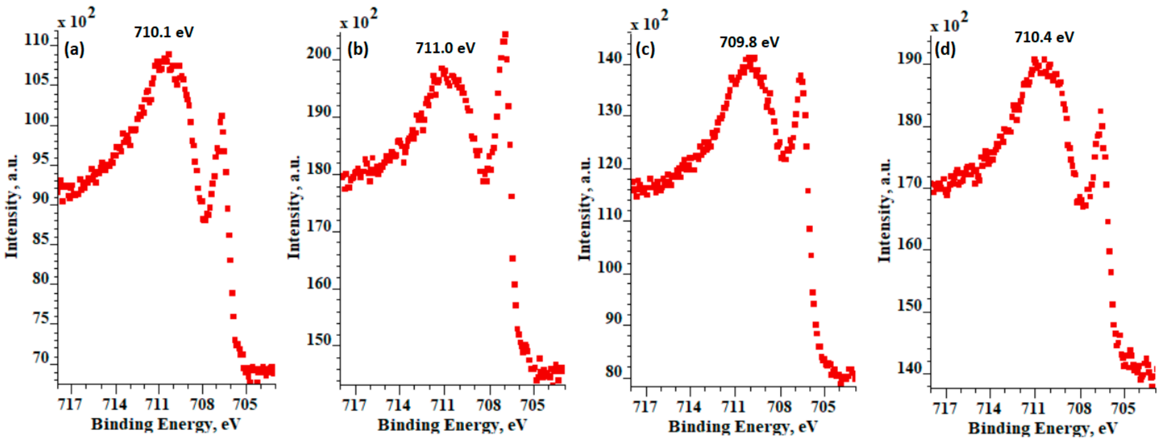

Figure S23. Iron Fe2p3/2 XPS spectra of the passive layer of electropolished stainless steels: (**a**) 18Cr21Mn2NiN, (**b**) 20Cr20Mn7Ni2MoN, (**c**) 18Cr15Ni3Mo, (**d**) 27Cr29Ni3Mo.

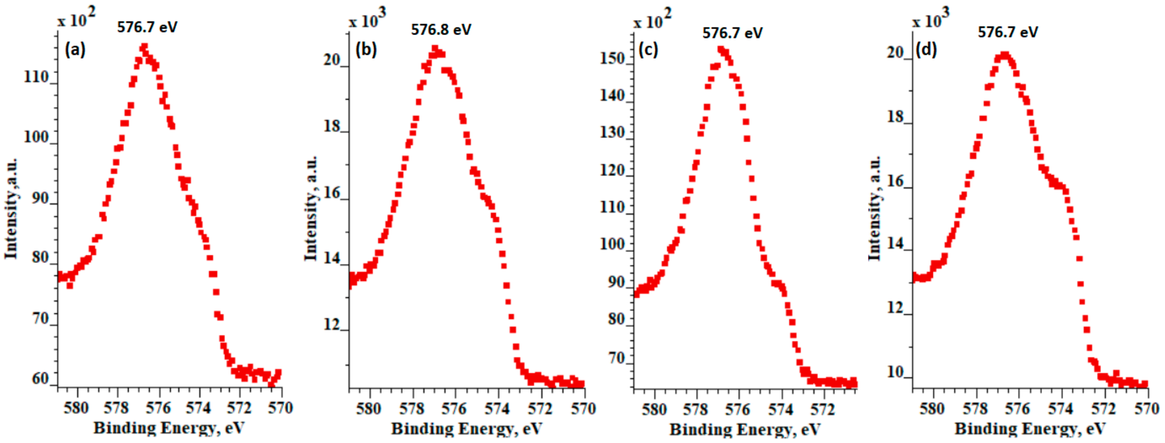

Figure S24. Chromium Cr2p3/2 XPS spectra of the passive layer of electropolished stainless steels: (**a**) 18Cr21Mn2NiN, (**b**) 20Cr20Mn7Ni2MoN, (**c**) 18Cr15Ni3Mo, (**d**) 27Cr29Ni3Mo

Table S1. Binding energies (BE, eV) of iron, chromium compounds.

	$Fe2p_{3/2}$	P2p	S2p	O1s		$Cr2p_{3/2}$	P2p	S2p	O1s
Fe^0	706.6–707	–	–	–	Cr^0	574.2–574.4	–	–	–
FeO	709.4	–	–	530.0	Cr_2O_3	576.4–576.8	–	–	530.2
Fe_2O_3	710.9	–	–	530.0	CrOOH	576.8–577.0	–	–	531.4
FeOOH	711.3–711.8	–	–	530.0	$Cr(OH)_3$	577.2–577.3	–	–	531.2
$FeSO_4$	711.0–712	–	169.1	532.2	CrO_3	579.2	–	–	530.1
$Fe_2(SO_4)_3$	713.4–713.5	–	–	531.8	CrO_4^{2-}	579.0–579.5	–	–	–
$FePO_4$	712.7	133.1	–	532.0	$Cr_2(SO_4)_3$	578.5	–	169.5	532.0
$Fe_3(PO_4)_2$	712.5	133.3	–	532.0	$CrPO_4$	577.9	133.7	–	–

Reference

1. NIST. ODI SRDATA Links. Available online: https://srdata.nist.gov/ (accessed on 31 July 2020)
